# *WDR36* Regulates Trophectoderm Differentiation During Human Preimplantation Embryonic Development Through Glycolytic Metabolism

**DOI:** 10.1002/advs.202412222

**Published:** 2024-12-10

**Authors:** Shiyu An, Shuyue Hou, Feifei Xu, Huanyu Yan, Wenyi Zhang, Jinfeng Xiang, Haoran Chen, Hanwen Zhang, Lingling Dong, Xiaobin Sun, Ran Huo, Yun Chen, Xi Wang, Yang Yang

**Affiliations:** ^1^ State Key Laboratory of Reproductive Medicine and Offspring Health Nanjing Medical University Nanjing Jiangsu 211166 China; ^2^ School of Pharmacy Nanjing Medical University Nanjing 211166 China; ^3^ Fourth Clinical Medicine College Nanjing Medical University Nanjing 210004 China; ^4^ Department of Obstetrics Women's Hospital of Nanjing Medical University Nanjing Maternity and Child Health Care Institute Nanjing 210004 China; ^5^ Department of Prenatal Diagnosis of the First Affiliated Hospital of Nanjing Medical University Nanjing 210029 China; ^6^ Innovation Center of Suzhou Nanjing Medical University Suzhou Jiangsu 215000 China

**Keywords:** glycolysis, human embryonic development, LDHA, trophectoderm lineage commitment, WDR36

## Abstract

Mammalian pre‐implantation development is a complex process involving sophisticated regulatory dynamics. WD repeat domain 36 (WDR36) is known to play a critical role in mouse early embryonic development, but its regulatory function in human embryogenesis is still elusive due to limited access to human embryos. The human pluripotent stem cell‐derived blastocyst‐like structure, termed a blastoid, offers an alternative means to study human development in a dish. In this study, after verifying that *WDR36* inhibition disrupted polarization in mouse early embryos, it is further demonstrated that *WDR36* interference can block human blastoid formation, dominantly hindering the trophectoderm lineage commitment. Both transcriptomics and targeted metabolomics analyses revealed that *WDR36* interference downregulated glucose metabolism. WDR36 can interact with glycolytic metabolic protein lactate dehydrogenase A (LDHA), thereby positively regulating glycolysis during the late stage of human blastoid formation. Taken together, the study has established a mechanistic connection between WDR36, glucose metabolism, and cell fate determination during early embryonic lineage commitment, which may provide potential insights into novel therapeutic targets for early adverse pregnancy interventions.

## Graphical abstract

WD repeat domain 36 (WDR36) is essential for early embryonic development. Using a human 8CLC‐derived blastoid model, this study demonstrates that inhibiting WDR36 disrupts trophectoderm commitment. Transcriptomic and metabolomic analyses reveal a downregulation of glucose metabolism in WDR36 interfered‐blastoid, linking WDR36 to glycolysis and cell fate determination. These findings highlight potential therapeutic targets for addressing early pregnancy complications.

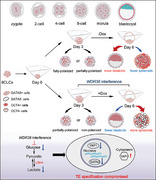

## 1 Introduction

Preimplantation development marks the initial stage of a new life. Starting from a fertilized zygote, successive cell cleavages lead to the formation of a blastocyst. This process is accompanied by several critical events, including cell polarization and compaction, and the cell differentiation into the trophectoderm (TE) and inner cell mass (ICM).^[^ ^1^ ^]^ Despite the increasing successful rate of in vitro fertilization (IVF) strategy in addressing infertility clinically, embryo quality remains a major contributing factor to the failure of IVF cycles. Clinical statistics indicate that ≈10% of fertilized eggs arrest during the cleavage stage of the preimplantation phase,^[^ ^2^ ^]^ with varied reasons for this developmental arrest. Therefore, it is imperative to decode the mechanisms underlying human embryonic arrest or degeneration to improve IVF success rates.

WD40 repeat (WDR) domain is the most abundant protein‐interaction domain in the human proteome, with more than 360 domains currently annotated. WDR proteins contribute to a wide array of cellular functions, such as cell division, cell fate determination, early embryonic development, transmembrane signaling, mRNA modification, and vesicle fusion.^[^ ^3^ ^]^ Notably, WDR36, a 105 kDa protein containing 14 WD40 repeats, likely folds into two β‐propellers. This protein was initially identified as a causative gene in primary open‐angle glaucoma (POAG), the most common subtype of glaucoma.^[^ ^4^ ^]^ Later, it is also found to express in various tissues, with high levels of its mRNA detected in the heart, skeletal muscle, pancreas, liver, and placenta.^[^ ^4^ ^]^ Studies have demonstrated the presence of *WDR36* transcripts at all embryonic stages,^[^ ^5^ ^]^ and targeted deletion of *WDR36* in mouse leads to abnormal preimplantation embryonic development, failing to form blastocysts.^[^ ^6^ ^]^ Mechanistic studies have identified WDR36 as functional homolog of yeast Utp21, an essential nucleolar ribonucleoprotein in the yeast *Saccharomyces cerevisiae*.^[^ ^7^ ^]^ Loss of WDR36 in zebrafish caused defects in 18S rRNA processing, disruption of nucleolar morphology, and activation of the P53 stress response pathway.^[^ ^5^ ^]^ Despite these findings, the mechanism by which WDR36 induces blastocyst degeneration in mouse is still unclear, and its role in early human embryonic development is yet to be determined.

The natural development of the human embryo occurs within the uterus in maternal body, posing significant challenges for mechanistic research. The limited availability of embryonic resources, the difficulty of embryo culture in vitro, and strict ethical guidelines seriously constrain research on human embryogenesis. Consequently, scientists have to heavily rely on experiments conducted in other mammals, especially mouse. Although embryonic development follows a broadly similar program across mammals, there are remarkable species‐specific differences.^[^ ^8^ ^]^ To address these challenges, multiple subtypes of human pluripotent stem cells (hPSCs) have been used to construct blastocyst‐like structures, known as blastoids, offering inexhaustible resources for modeling human embryogenesis.^[^ ^9^ ^]^ Notably, Yu et al.^[^ ^10^ ^]^ and Mazid et al.^[^ ^11^ ^]^ have described a transgene‐free, rapid, and controllable method for producing 8C‐like cells (8CLCs) from hPSCs, which can produce both embryonic and extraembryonic lineages in vitro or in vivo in the form of blastoids. These blastoids, which closely resemble natural embryos in many aspects, including morphology, lineage composition and localization, and transcriptome, can serve as invaluable models for studying human early embryonic development in vitro.

In this study, we found that *WDR36* interference compromises the polarization of mouse morula, culminating in blastocyst degeneration. Concurrently, we explored the effects of *WDR36* on human early embryonic development based on human 8CLCs‐derived‐blastoid model. We found that *WDR36* interference hinders human blastoid formation, primarily affecting the TE lineage commitment. Transcriptomic analysis revealed that *WDR36* interference leads to downregulation of genes implicated in TE lineage differentiation and glucose metabolism. Additionally, mass spectrometry detection assay demonstrated a reduction in glucose and lactate production upon *WDR36* interference. Finally, WDR36 can interact with metabolic enzymes associated with glycolysis and positively regulates glycolysis levels during the late stage of human blastoid development. This study not only deciphers the role of *WDR36* in regulating cell differentiation during early human embryonic development, but also underscores the feasibility of the blastoid model as a viable alternative to natural human blastocysts for investigating human embryogenesis and early developmental defects.

## 2 Results

### 2.1 Inhibition of *WDR36* Impairs the Polarization During Both Mouse Embryo Development and Human Blastoid Formation

Given the easy availability of mouse embryos, we first studied the function of *WDR36* on mouse embryonic development. We generated siRNA specific against mouse *WDR36* mRNA and injected it into the cytoplasm of 0.5 dpc mouse zygotes (0 h). During the first 36 h, mouse embryos in all three groups (Not‐injected, Non‐target, and si‐*WDR36*) developed normally, with the developmental kinetics of Not‐injected and si‐WDR36 injected embryos slowing down slightly. At 54 h, a small number of compacted embryos had already appeared in the not‐injected and non‐target siRNA injected groups, while none were observed in the interference group. By 70 h, most embryos in the not‐injected and non‐target siRNA injected groups had reached the morula stage. In contrast, more than one‐third of the embryos in the interference group remained at the 3–4 cell stage (Figure S1A‐B, Supporting Information). This phenomenon could be attributed to the sharp decrease in WDR36 protein expression, which also verified the efficacy of the siRNA interference (Figure S1C‐D, Supporting Information). At 100 h, only a small fraction of embryos in the si‐*WDR36* group progressed to the blastocyst stage, with a quite high proportion either degenerating or remaining at the morula stage (Figure S1A‐B, Supporting Information). Summarily, the disruption of *WDR36* led to delayed embryonic development from the 2–4 cell stage onward, with degeneration primarily occurring during the transition from the morula to blastocyst stage, thereby significantly reducing the blastocyst development rate, consistent with the findings of Martin et al.^[^ ^6^ ^]^ Given the fact that the process of compaction and apical‐basal polarization triggers a series of events during mammalian development, we further examined the effects of *WDR36* on embryonic polarization and compaction. The immunofluorescent analysis indicated that the expression of ZO‐1 was not affected by *WDR36* interference, suggesting that compaction remained normal (Figure S1E, Supporting Information). Following compaction, polarization begins, with the apical domain protein EZRIN starting to accumulate at the apical side of the blastomeres.^[^ ^12^ ^]^ Notably, while all embryos in the non‐injected and non‐target siRNA injected groups were fully polarized, ≈30% of si‐*WDR36* embryos exhibited a partially‐polarized pattern (Figure S1F‐G, Supporting Information). Together, these results imply that *WDR36* plays a role in the polarization of mouse embryos, and the deficiency of *WDR36* affects embryo polarization and leads to blastocyst degeneration, but does not affect compaction.

**Figure 1:**
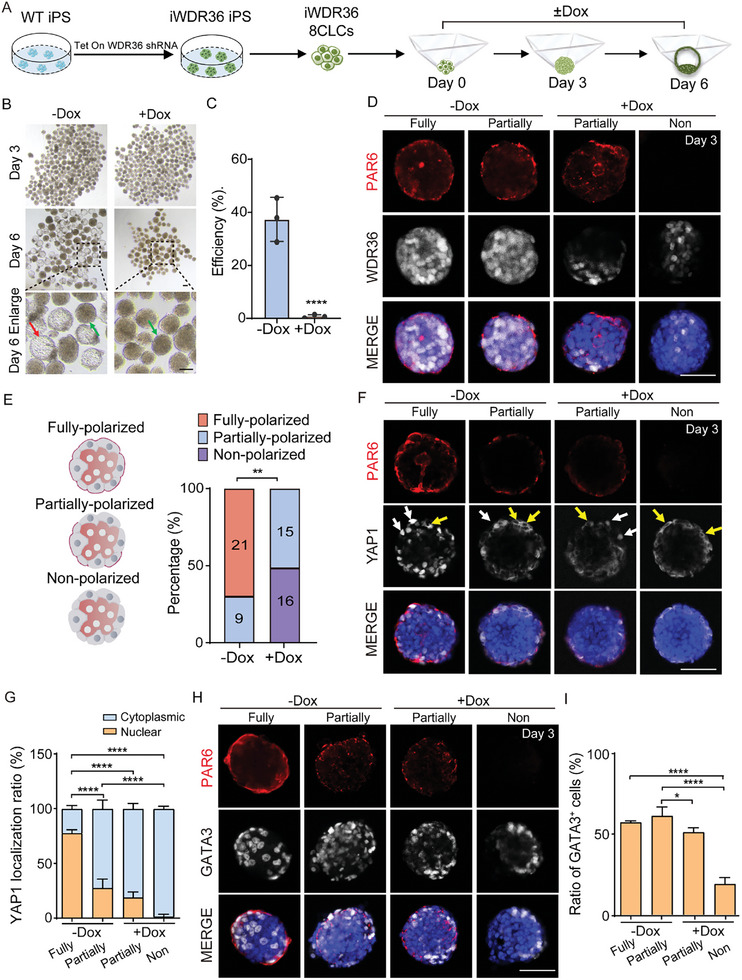
Inhibition of *WDR36* throughout the entire blastoid formation process disrupts the polarization potential. A) Flow chart of using human blastoid model to investigate effects of *WRD36* on human blastocyst development. B) Phase‐contrast images of ‐Dox and +Dox groups cultured in the indicated medium conditions. The enlarged image in the lower pannel showed spheroids (green arrow) and blastoids (red arrow) on day 6. Bars = 100 µm. C) Quantification of iPS‐8CLCs‐derived blastoid formation efficiency in the assay described in (A). Data were represented as mean ± S.D. in three independent experiments and two‐tailed Student's *t*‐test were used for statistical analysis. ****p < 0.0001. D) Representative immunofluorescence co‐staining images of PAR6 and WDR36 in day 3′s aggregates, with and without *WDR36* interference. Bar = 50 µm. E) Percentages of fully polarized, partially‐polarized and non‐polarized cell aggregates in each group. The number in each bar indicates the number of cell aggregates of the related category. Data were represented three independent experiments and Fisher's exact test were used for statistical analysis. **p < 0.01. F) Representative immunofluorescence co‐staining images of PAR6 and YAP1 in day 3′s aggregates, with and without *WDR36* interference. The representative images selected were collected under the laser confocal microscopy and presented in a single‐plane format. Bar = 50 µm. White arrows illustrate the cells with nuclear YAP1 localization while yellow arrows highlight cells with cytoplasmic YAP1. G) Average ratios of cytoplasmic and nuclear localization of YAP1 in day 3′s aggregates. Data were represented as mean ± S.D. in three independent experiments and one‐way ANOVA were used for statistical analysis. ****p < 0.0001. H) Representative immunofluorescence co‐staining images of PAR6 and GATA3 in day 3′s aggregates, with and without *WDR36* interference. Bar = 50 µm. I) Proportions of GATA3^+^ cells in day 3′s aggregates. Data were represented as mean ± S.D. in three independent experiments and one‐way ANOVA were used for statistical analysis. *p < 0.05, ****p < 0.0001. No labeling indicates no statistical significance.

To further explore the role of *WDR36* in the development of human embryos, we continue to explore its impact using human stem cell‐derived blastoid.^[^ ^10^ ^]^ We constructed a Dox‐induced *WDR36* knockdown sub cell line based on iPS cells, referred to as iWDR36 iPS. Upon adding Dox, severe clonal differentiation was observed (Figure S2A, Supporting Information), consistent with our previous report.^[^ ^13^ ^]^ qRT‐PCR and immunofluorescence analysis confirmed the successful construction (Figure S2B‐D, Supporting Information). Initially, we converted the primed iWDR36 iPS into dome‐shaped 8CLCs in the 8CLCs medium. Then, we aggregated iPS‐derived 8CLCs in the AggreWell plate according to the protocol of Harunobu et al.,^[^ ^9e^ ^]^ counting as day 0, and continuously treated cells with (+Dox group) or without Dox (‐Dox group) from that point for 6 days (**Figure** **1**A). By day 3, no obvious morphological differences were noted. However, by day 6, the *WDR36* interference group did not form cavity‐like features, remaining as spheroid, while ≈40% of the aggregates in the control group developed cavity‐like features, which were designated as blastoid (Figure 1B‐C). For the sake that results derived from stem cell studies might vary across different cell lines, we conducted additional experiments using the hEPS1 cell line, which originated from the human blastocyst rather than being reprogrammed from somatic cells, to strengthen the validity of our findings. As expected, when hEPS1‐derived 8CLCs were used as surrogates, no cavity‐like blastoid structures formed following *WDR36* interference (Figure S3A‐B, Supporting Information).

Given the above results from mouse natural embryos, we then examined the compaction and polarization in the day 3 aggregates. By immunofluorescence analysis, we found that, similar to the results in mice, there was no significant difference in the expression of ZO‐1 protein between groups with and without *WDR36* interference (Figure S4A, Supporting Information), suggesting that *WDR36* interference did not affect embryo compaction. While EZRIN has been established as an apical marker in mouse embryos,^[^ ^14^ ^]^ PAR6 is well‐documented in human embryos by Zhu et al.^[^ ^15^ ^]^ The results showed that both of two groups had partially‐polarized aggregates. Unlike natural mouse embryos, a large number of non‐polarized aggregates emerged after *WDR36* interference, and these aggregates completely lacked the expression of PAR6 protein. Furthermore, none fully‐polarized aggregates were observed after *WDR36* interference (Figure 1D‐E). Studies have demonstrated that the active YAP1 is confined by the presence of the polar region, and its nuclear activity leads to the upregulation of GATA3 expression during human embryo compaction.^[^ ^16^ ^]^ Therefore, we further examined the expression of active YAP1 and GATA3. Our findings revealed that ≈75% of outer cells displayed high levels of nuclear YAP1 (active YAP1^+^) in the fully‐polarized aggregates of the ‐Dox group. The proportion of active YAP1^+^ cell markedly decreased in the partially‐polarized aggregates of both the ‐Dox and +Dox groups. This reduction was even more pronounced in the non‐polarized aggregates of the +Dox group, where most of the peripheral cells had YAP1 remaining in the cytoplasm (active YAP1^−^) (Figure 1F‐G). Concurrently, both fully‐polarized and partially‐polarized aggregates in the ‐Dox group exhibited a high proportion of GATA3^+^ cells. However, following *WDR36* interference, the proportion of GATA3^+^ cells decreased significantly, especially in non‐polarized aggregates (Figure 1H‐I). Additionally, we conducted a 2D trophoblast differentiation assay using 8CLCs. The 8CLCs were seeded in hTSC medium^[^ ^10^ ^]^ and cultured for 5 days. Throughout the process, no Dox was added to the control group, whereas in the experimental group, Dox was included. As shown Figure S4B (Supporting Information), in the ‐Dox group, a large number of GATA3^+^ cells were observed, along with substantial nuclear translocation of YAP1, which co‐localized with GATA3. There was also a minor presence of YAP1 in the cytoplasm of cells that were GATA3^−^. Conversely, in the +Dox group, no GATA3^+^ cells were found, and YAP1 remained in the cytoplasm (Figure S4B, Supporting Information). These findings combinely suggest that the disruption of *WDR36* mainly affects YAP1 activation, ultimately influencing the initiation of TE lineage specification.

In summary, these findings demonstrate that *WDR36* interference can lead to abnormal polarization during processes of mouse embryo development and human blastoid formation, with no impact on compaction. Moreover, *WDR36* interference affects the initiation of TE lineage specification during blastoid formation on day 3.

### 2.2 Inhibition of *WDR36* Affects Cell Lineage Specification During Human Blastoid Formation

**Figure 2:**
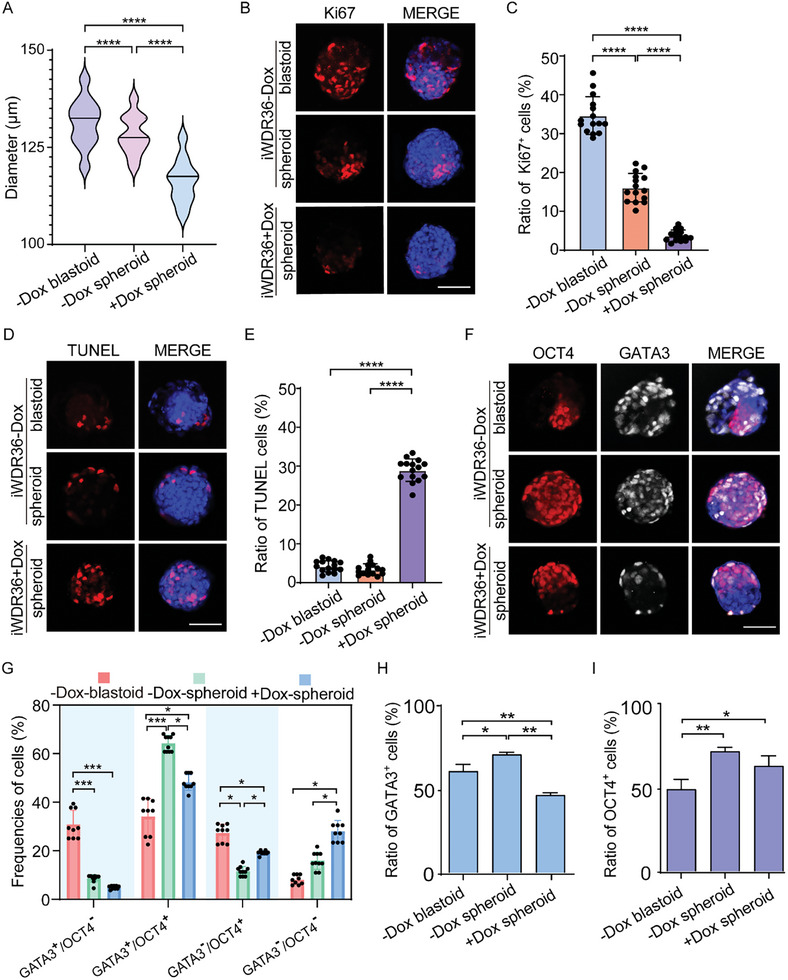
Interference with *WDR36* throughout the process disrupted blastoid formation of human iPS‐derived 8CLCs. A) Quantification of the diameters of iPS‐8CLCs‐blastoids and iPS‐8CLCs‐spheroids. B) Immunofluorescent images of the blastoids and spheroids to visualize proliferation (Ki67). Bar = 100 µm. C) Proportion of Ki67 positive cells (Ki67^+^ cell number/total cell number) in each blastoid or spheroid. D) Immunofluorescent images of the blastoids and spheroids to visualize apoptosis (TUNEL). Bar = 100 µm. E) The apoptotic ratio (apoptotic cell number/total cell number) in each blastoid or spheroid. F) Immunostaining for lineage‐specific markers in the blastoids and spheroids (GATA3 for TE, OCT4 for ICM). Bar = 100 µm. G) Frequencies of different cell types in each blastoid/spheroid. (H‐I) Percentages of GATA3^+^ and OCT4^+^ cells from (G). In each group, 15 iPS‐blastoids or spheroids were counted. Data were represented as mean ± S.D. in three independent experiments and one‐way ANOVA were used for statistical analysis. *p < 0.05, **p < 0.01, ***p < 0.001, ****p < 0.0001. No labeling indicates no statistical significance.

Next, we investigated whether *WDR36* interference also affects blastoid on day 6, by continously culturing 8CLCs in the AggreWell plate in blastoid induction medium togethor with (+Dox group) or without Dox (‐Dox group) treatment. Bright‐field images in Figure 1B suggests that the diameters of blastoids and spheroids on day 6 may differ. As expected, the average diameters of spheroids in the ‐Dox group were significantly smaller than those of blastoids, while the average diameters of spheroids in the +Dox group were even smaller (**Figure** **2**A; Figure S5A, Supporting Information). Consequently, we further examined proliferation and apoptosis. As shown in Figure 2B‐C, spheroids in both the ‐Dox and +Dox groups displayed a dramatic reduction in Ki67 protein, with the +Dox group experiencing a particularly pronounced decrease, resulting the ratio of Ki67^+^ cell less than 5% (Figure 2B‐C). Simultaneously, TUNEL assay revealed that apoptosis markedly increased in spheroids subjected to *WDR36* interference. In contrast, no significant difference in apoptosis rates was observed between blastoids and spheroids in the ‐Dox group, both of which maintained relatively low apoptosis levels (Figure 2D‐E). Similar effects were noted in groups originated from hEPS1‐derived 8CLCs (Figure S5B‐C, Supporting Information). The above results suggest that interfering with *WDR36* affects the proliferation and apoptosis of blastoids and spheroids, potentially contributing to the decreased efficiency of blastoid formation.

Given that *WDR36* interference affects the initiation of TE lineage specification on day 3 (Figure 1H‐I), we subsequently deciphered the cell lineage composition of blastoids and spheroids on day 6. The expression patterns of OCT4 and GATA3 in the ‐Dox group blastoids demonstrated a cellular composition consistent with that of human natural blastocysts. It seemed that there were more GATA3^+^ and OCT4^+^ cells in the spheroids of the ‐Dox group, whereas spheroids of the +Dox group had much fewer GATA3^+^ cells (Figure 2F; Figure S5D, Supporting Information). Next, we conducted a more detailed statistical analysis.

The results showed that, in comparison to blastoids in the ‐Dox group, the proportions of GATA3^+^/OCT4^−^ and GATA3^−^/OCT4^+^ cells significantly decreased following *WDR36* interference, whereas the proportion of GATA3^+^/OCT4^+^ and GATA3^−^/OCT4^−^ cells were significantly increased (Figure 2G), which were consistent with those observed in balstoids/spheroids derived from hEPS1‐8CLCs (Figure S5E, Supporting Information). These alternations collectively led to a substantial decrease in the proportion of GATA3^+^ cells and a significant increase in the proportion of OCT4^+^ cells (Figure 2H‐I). A similar changing trend was also noted in the spheroids of the +Dox group derived from hEPS1‐originated 8CLCs, with a slight variation in the trend regarding the proportion of OCT4^+^ cell (Figure S5F‐G, Supporting Information). The above results suggest that the inability to form proper blastoid structures following *WDR36* interference may be attributed to deficient TE lineage commitment.

We also analyzed differences between spheroids in the ‐Dox group and those in the *WDR36* interference group. Compared to control spheroids, the proportion of GATA3^+^/OCT4^+^ cells significantly decreased upon *WDR36* interference, while those of GATA3^−^/OCT4^+^ and GATA3^−^/OCT4^−^ cells significantly increased (Figure 2G). A similar trend was observed in spheroids derived from hEPS1‐originated 8CLCs, with an increase in the proportion of GATA3^−^/OCT4^−^ cells, although this increase was not statistically significant (Figure S5E, Supporting Information). Consequently, despite both being spheroid structures, *WDR36* interference leads to a significant decrease in the proportions of both GATA3^+^ and OCT4^+^ cells within the spheroids, implying a more severe disturbance in cell fate determination.

Collectively, our findings suggest that *WDR36* interference impairs the generation of human blastoid and primarily affects the cell lineage specification, particularly toward the TE lineage.

### 2.3 *WDR36* is Indispensable During Late Stages of Human Blastoid Formation

**Figure 3:**
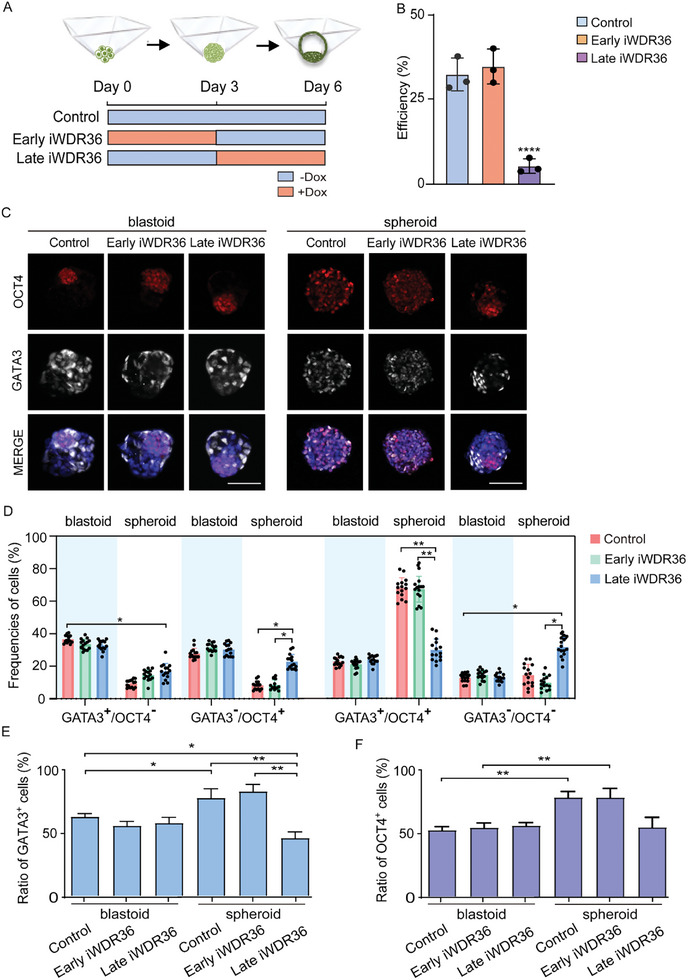
*WDR36* interference mainly hinders the late stage of iPS‐8CLCs‐blastoids formation. A) A flowchart showing the experiment design to determine the main influencing period of *WDR36* during the iPS‐8CLCs‐blastoid self‐organization. B) Quantification for iPS‐8CLCs‐blastoid formation efficiencies of the early and late *WDR36* interference groups. C) Immunostaining for lineage‐specific markers in blastoids/spheroids of Dox treated and untreated groups. Bar = 100 µm. D) Frequencies of different cell types in each blastoid or spheroid. E‐F) Percentages of GATA3^+^ and OCT4^+^ cells from (D). In each group, 15 iPS‐8CLCs‐blastoids or spheroids were counted. Data were represented as mean ± S.D. in three independent experiments and one‐way ANOVA were used for statistical analysis. *p < 0.05, **p < 0.01, ***p < 0.001, ****p < 0.0001. No labeling indicates no statistical significance.

To address the main stage at which *WDR36* influences human blastoid formation, we inhibited WDR36's expression at different time periods by treating cells with Dox, as illustrated in **Figure** **3**A. The statistical results showed that inhibiting *WDR36* from day 3 onward (Late iWDR36) dramatically impaired the blastoid formation potential (Figure 3B), aligning with the effects observed when WDR36 was inhibited throughout the entire process (Figure 1C; Figure S3B, Supporting Information). Conversely, early‐stage interference with *WDR36* (Early iWDR36) did not have an obvious effect on human blastoid formation, whether utilizing iPS‐derived or hEPS1‐derived 8CLCs (Figure 3B; Figure S6A, Supporting Information). Therefore, we concluded that *WDR36* mainly hinders human blastoid formation at the later stage.

Given that *WDR36* interference led to disturbance of cell fate determination (Figure 2F‐I; Figure S6D‐G, Supporting Information), we proceeded to analyze whether inhibiting *WDR36* at the later stage would yield comparable effects. First, we compared blastoids in the three groups (Early iWDR36, Late iWDR36 and ‐Dox Control), though blastoids obtained in the Late iWDR36 group were much fewer. Immunofluorescent and statistical results showed that all the blastoids in the three groups had similar cellular composition (Figure 3C‐D; Figure S6B‐C, Supporting Information).

Notably, the ratio of GATA3^+^/OCT4^+^ of spheroids in Late iWDR36 group, which was comparable to those of blastoids in all the three groups, exhibited a significant downward trend when compared to spheroids in both the Control and Early iWDR36 groups (Figure 3D; Figure S6C, Supporting Information). By contrast, spheroids in Late iWDR36 group had significantly higher ratios of GATA3^−^/OCT4^+^ and GATA3^−^/OCT4^−^ cells compared to spheroids in the Control and Early iWDR36 groups (Figure 3D; Figure S6C, Supporting Information). Concurrently, the ratio of GATA3^+^/OCT4^−^ spheroids was remarkably decreased relative to those of blastoids. These trends combinedly cumulated in a significant reduction in ratio of GATA3^+^ cells in spheroids in the Late iWDR36 group (Figure 3E; Figure S6D, Supporting Information), while the ratio of OCT4^+^ cells remained unchanged (Figure 3F; Figure S6E, Supporting Information). This observed phenomenon is aligned with the effects observed when *WDR36* was inhibited throughout the entire process.

In general, by switching the medium between +Dox and ‐Dox at various time points, we identified a critical period for *WDR36* requirements occurring within a 3‐day window between day 4 and day 6. Additionally, we concluded that *WDR36* plays an important role in the late development of human blastoids and may regulate the fate decision of the TE lineage.

### 2.4 Transcriptome Analysis Further Demonstrates that *WDR36* Interference Mainly Regulates TE Lineage Differentiation

To investigate the mechanism by which *WDR36* affects blastoid formation, we collected samples from the Control and Late iWDR36 groups on day 4 and day 6 for RNA sequencing analysis (Figure S7A, Supporting Information). Initially, we compared the transcriptomics of the day 6 Control blastoids with those of human natural blastocysts^[^ ^17^ ^]^ and other reported blastoids available from published datasets.^[^ ^9^, ^18^ ^]^ At the lineage level, principal component analysis (PCA) revealed that our control blastoids closely resembled human natural blastocysts and were also similar to the blastoids reported by others (Figure S7B, Supporting Information). Furthermore, we found that blastoids in the day 6 Control group exhibited comparable expression levels of lineage‐specific marker genes to those of blastocysts (Figure S7C, Supporting Information). Overall, these results demonstrate that the blastoids in this study closely resemble human natural blastocyst at the transcriptional level.

**Figure 4:**
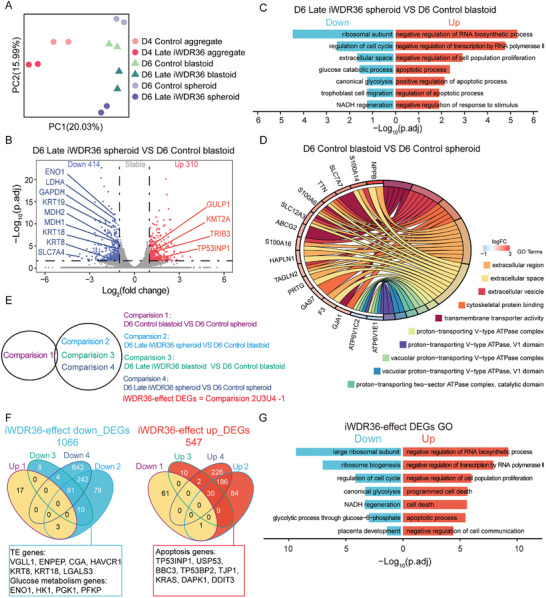
Transcriptome analysis shows that *WDR36* interference mainly affects TE lineage differentiation in the late development of human blastoid. A) Plot of the principal component analysis (PCA) of the Control and Late iWDR36 groups on day 4 and day 6. B) Volcano plots for the globally transcriptional differences between day 6 Late iWDR36 spheroid and day 6 Control blastoid. Red dots indicated up‐regulated (FC > 2) and blue dots indicated down‐regulated (FC < −2) genes with adjusted *p* < 0.05. Grey dots indicated genes with no significant difference with either adjusted *p* ≥ 0.05 or absolute value of FC ≤ 2. C) Enrichment of GO terms of up‐regulated (red) and down‐regulated (blue) genes in the comparison of D6 Late iWDR36 spheroid and D6 Control blastoid. D) Chord plot showed enriched GO terms of up‐regulated genes and down‐regulated genes in the comparison of D6 Control blastoid and D6 Control spheroid. E) Identification strategies for candidate genes contributing to the failure of blastoid development induced by WDR36. F) Venn diagram showing the common set of 1066 down‐ and 547 up‐regulated genes identified at the overlap of multiple comparative transcriptome data. G) Enrichment of GO terms of up‐regulated (red) and down‐regulated (blue) genes in iWDR36‐effect DEGs. D4 means day 4, D6 means day 6.

Additionally, consistent with the aforementioned immunofluorescence results (Figure 3C), transcriptome analysis indicated that the few blastoids in the Late iWDR36 group were quite similar to the control blastoids (**Figure** **4**A). These blastoids exhibited comparable expression levels of lineage‐specific genes for the EPI (*POU5F1* and *SOX2*), HYPO (*GATA6* and *PDGFRA*), and TE (*GATA3* and *KRT8*), alongside only 103 differentially expressed genes (DEGs) down‐regulated and 51 DEGs up‐regulated (Figure S8A, Supporting Information). Gene Ontology (GO) analysis revealed that the down‐regulated genes were predominantly enriched in the regulation of cellular component organization, whereas the up‐regulated genes were enriched in endocytic vesicle (Figure S8B, Supporting Information). This correlates with the observation that a very small portion of aggregates, following *WDR36* interference, were able to form blastocyst‐like structures with same morphology and lineage differentiation as the Control group.

Subsequently, we analyzed the differences between spheroids in the Late iWDR36 group and control group blastoids, capturing both the effects due to *WDR36* interference and those inherent to the blastoid formation itself. We identified 310 up‐ and 414 down‐regulated genes (Figure 4B). As expected, many TE lineage genes, such as *KRT8*, *KRT18* and *KRT19*, were obviously down‐regulated. Meanwhile, apoptosis‐related genes, including *TRIB3*, *GULP1* and *TP53INP1*, were significantly up‐regulated in spheroids from the day 6 Late iWDR36 group (Figure 4B). GO analysis revealed that these down‐regulated genes were predominantly enriched in processes related to the cell cycle, its regulation, and trophoblast cell migration, while the up‐regulated genes were enriched in apoptotic process (Figure 4C). These results are also consistent with the previously observed significant increase in the proportion of TUNEL‐positive cells when *WDR36* was inhibited throughout the entire process (Figure 2D‐E; Figure S5B‐C, Supporting Information). Our transcriptome analysis also revealed that a marked downregulation of TE lineage‐related genes (*CGA* and *VGLL1*) in the comparison of D6 Late iWDR36 spheroid and D6 Control spheroid (Figure S8C, Supporting Information). The down‐regulated genes were predominantly enriched in ribonucleoprotein complex biogenesis and cell cycle (Figure S8D, Supporting Information), which are related to TE lineage differentiation.^[^ ^19^ ^]^

To resolve the inherent limitations associated with the blastoid formation system itself, we conducted a DEG analysis comparing Control blastoids to Control spheroids. This analysis illustrated that these down‐regulated genes were principally enriched in the proton‐transporting V‐type ATPase complex, whereas up‐regulated genes were enriched in the extracellular space (Figure 4D). Then, by excluding DEGs that might be attributed to the blastoid formation process, we identified those specifically induced by *WDR36* interference, comprising 1066 down‐ and 547 up‐ regulated DEGs (Figure 4E‐F). GO analysis revealed that these down‐regulated DEGs were chiefly enriched in the regulation of the cell cycle and placenta development, while the up‐regulated genes were enriched in programmed cell death, cell death and apoptotic process (Figure 4G).

To determine whether deficient TE lineage commitment caused by *WDR36* interference started at an earlier formation stage, we analyzed day 4 aggregates from both the Late iWDR36 group and the Control group. An overall of 261 DEGs were identified between these groups, with significant downregulation of several TE lineage‐specific genes, such as *GATA2*, *GATA3*, and *NR2F2*, upon *WDR36* interference (Figure S9A, Supporting Information). GO analysis revealed that these down‐regulated genes were predominantly enriched in categories related to cell differentiation involved in embryonic placenta development, placenta development, embryo development and cell population proliferation (Figure S9B, Supporting Information). Furthermore, global gene expression profiling and gene set enrichment analyses (GSEA) demonstrated that *WDR36* interference repressed placenta development and developmental cell growth in day 4 aggregates (Figure S9C‐D, Supporting Information). These findings indicate that the regulatory effect of *WDR36* on the TE lineage can be detected as early as 24 h post‐interference.

Taken together, our results suggest that *WDR36* interference mainly regulates the differentiation of TE lineage at the later stage of blastoid formation.

### 2.5 *WDR36* Interference Disturbs Glucose Metabolism at the Late Stage of Blastoid Formation

Decades of research have underscored the critical role of metabolic homeostasis in preimplantation development, with glucose metabolism specifically influencing the fate of TE cells during mammalian embryogenesis.^[^ ^20^ ^]^ The above DEG analyses revealed that *WDR36* interference caused a significant downregulation of genes associated with glucose metabolism (e.g., *HK1*, *ENO1*, *GAPDH*) in spheroids (Figure 4B,F; Figure S8C, Supporting Information). Further GO analysis also revealed that DEGs were mainly enriched in pathways involving glucose catabolic process, glycolytic process through glucose‐6‐phosphate, and NADH regeneration (Figure 4C,G; Figure S8D, Supporting Information). In the process of glycolysis, the phosphorylation of glucose to glucose 6‐phosphate (G6P) by hexokinases (HKs) is the initial step of glucose metabolism. NADH, produced in subsequent reactions, acts as a carrier of biological hydrogen, aiding the transfer of electrons and hydrogen, which in turn promotes energy conversion and utilization.^[^ ^21^ ^]^ The above results suggest that *WDR36* may regulate glucose metabolism, subsequently affecting blastoid formation.

**Figure 5:**
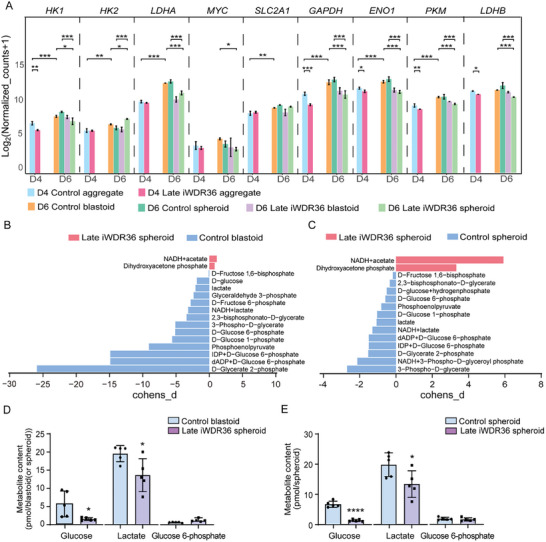
WDR36 modulates glucose metabolism during late developmental stage of blastoid. A) The expression levels of representative genes for glycolysis in D4 aggregate and D6 blastoid/spheroid. We mainly present the following statistical analysis results: D4 Late iWDR36 aggregate VS D4 Control aggregate, D6 Control blastoid VS D4 Control aggregate, D6 Late iWDR36 spheroid VS D6 Control blastoid and D6 Late iWDR36 spheroid VS D6 Control spheroid. Data were represented as mean ± S.D. in three independent experiments and Wald test was used for statistical analysis. *p < 0.05. **p < 0.01. ***p < 0.001. B‐C) Compass‐score differential activity test between *WDR36* interference and control group in day 6. D–E) Glucose, lactate, and glucose 6‐phosphate production in *WDR36* interference and control groups. Data were represented as mean ± S.D. in five independent experiments and two‐tailed Student's *t*‐test were used for statistical analysis. *p < 0.05. ****p < 0.0001. No labeling indicates no statistical significance. D4 means day 4, D6 means day 6.

During the in vivo development of human preimplantation embryos, glycolytic activity progressively increases starting from the cleavage stage.^[^ ^22^ ^]^ In our study of human blastoid formation, we observed a similar changing tendency for glycolysis‐associated genes, such as *HK1*, *LDHA*, *GAPDH, ENO1*, and *PKM*, consistent with human preimplantation embryos (**Figure** **5**A). However, this trend was reversed upon interference with *WDR36*. Within the first 24 h of *WDR36* interference, glycolysis‐related genes, including *HK1*, *GAPDH, ENO1*, and *PKM* were down‐regulated, whereas some genes, such as *HK2*, *LDHA*, *MYC*, and *SLC2A1*, remained unchanged (Figure 5A). Prolonged interference with *WDR36* intensified this downregulation, leading to further decreases in the expression levels of genes such as *LDHA, MYC*, and *ENO1* (Figure 5A). These findings suggest a more severe inhibition of glycolysis‐related genes with extended *WDR36* interference during the latter stage of blastoid formation.

Computational methods such as Compass have been developed to estimate metabolic flux from RNA‐seq data.^[^ ^23^ ^]^ Analysis using Compass further highlighted differences in glycolysis metabolism between the *WDR36* interference and control groups. The algorithm predicted that glycolytic reactions were generally more active in the control group compared to the *WDR36* interference group, in both spheroids and blastoids (Figure 5B‐C; Data S1, Supporting Information). Subsequently, we detected the concentrations of glycolysis‐related products through mass spectrometry (MS) and found that *WDR36* interference caused the concentrations of glucose and lactate to be significantly reduced compared to the control group, both in blastoids or spheroids, while the concentration of glucose 6‐phosphate remained unchanged (Figure 5D‐E). Therefore, these results further confirm that the interference of *WDR36* disturbed glucose metabolism during blastoid formation.

In summary, these findings indicate that *WDR36* deficiency affects glucose metabolism and ultimately hinders the late self‐organization of blastoids.

### 2.6 *WDR36* Regulates Glycolysis Activity in Human Blastoids Through Interacting with Glycolysis‐Associated Enzymes

**Figure 6:**
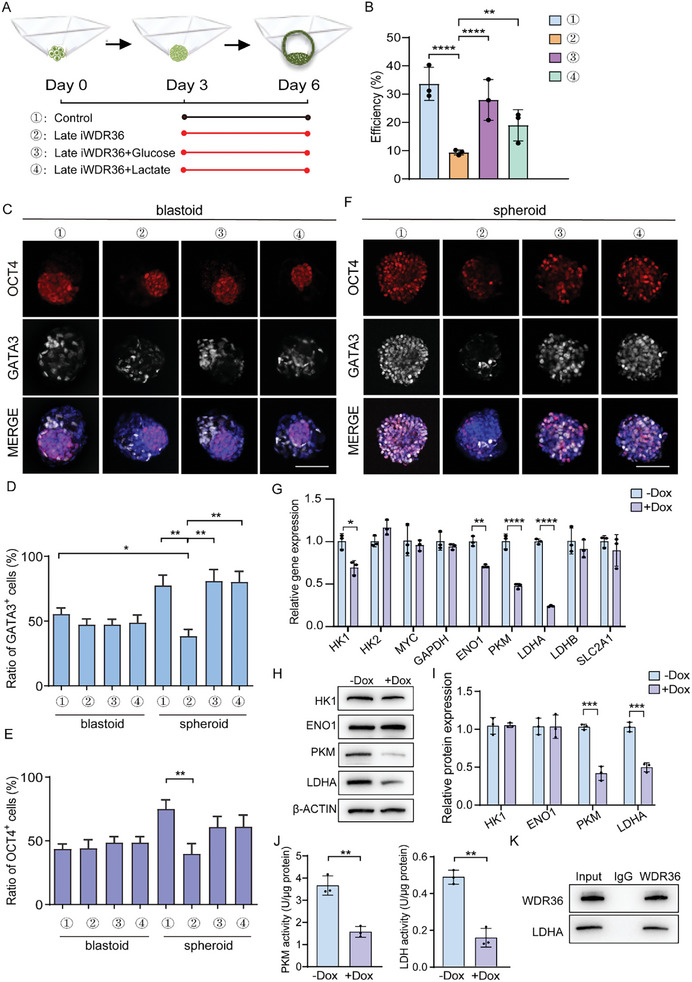
WDR36 regulates glycolysis metabolism in human blastoid through interacting with glycolysis‐associated enzymes. A) Schemes of the treatment period with Dox, glucose, or lactate during the iPS‐blastoid self‐organization. B) Quantification of iPS‐blastoids formation efficiency in the assay described in (A). C) Immunostaining for lineage‐specific markers in blastoids in the groups described in (A). Bar = 100 µm. D–E) Quantification of the percentage of GATA3^+^ or OCT4^+^ cells in iPS‐blastoid/spheroid. *N* = 15 iPS‐blastoids or spheroids. Data in B, D, and E were represented as mean ± S.D. in three independent experiments and one‐way ANOVA were used for statistical analysis. *p < 0.05. **p < 0.01, ****p < 0.0001. No labeling indicates no statistical significance. F) Immunostaining for lineage‐specific markers in spheroids in the groups described in (A). Bar = 100 µm. G‐J) The expression of genes involved in glycolysis with *WDR36* interference was determined using qRT‐PCR (G) and Western blot (H–I). (J) Activities of the glycolytic enzymes LDH and PKM with *WDR36* interference. Data in G, I, and J were represented as mean ± S.D. in three independent experiments and two‐tailed Student's *t*‐test was used for statistical analysis. * *P* < 0.05. (K) The interaction between WDR36 and glycolytic enzymes LDHA were demonstrated by co‐IP. ①, ‐Dox, ②, +Dox, ③, +Dox+Glucose, ④, +Dox+Lactate.

To elucidate the molecular mechanisms by which *WDR36* influences glucose metabolism, thereby affecting blastoid development, we first evaluated whether glucose and lactate supplementation could mitigate the effects of *WDR36* inhibition on blastoid developmental potential (**Figure** **6**A). Initially, there were no significant differences in the diameters of day 3′s aggregates among the four groups (Figure S10A‐B, Supporting Information). However, by day 6, the average diameter of the spheroids in the +Dox group had significantly decreased, whereas the diameters of the spheroids supplemented with glucose or lactate were comparable to that of the blastoids in the control group (Figure S10C‐D, Supporting Information). Furthermore, our data demonstrated that supplementing with glucose or lactate significantly restored the efficiencies of blastoid formation (Figure 6B). Immunofluorescent and statistical analysis also indicated no differences in lineage differentiation among the four groups of blastoids (Figure 6C‐E). In line with our previous observations, *WDR36* knockdown markedly reduced the population of GATA3^+^ cells in spheroids (Figure 6C‐D,F). However, co‐treatment with Dox and either glucose or lactate increased the proportion of GATA3^+^ cells. At the same time, the addition of glucose and lactate partially restored the proportion of OCT4^+^ cells, although this increase was not statistically significant (Figure 6C‐F).

We subsequently sought to investigate whether *WDR36* modulates blastocyst development by regulating glycolysis‐related enzymes. According to our previous transcriptome data (Figure 5A), we cultured the iPS‐8CLCs in PALLY medium, which is used for blastoid generation, and treated cells with or without Dox for 72 h simultaneously, to validate the expression levels of additional glycolysis‐related genes within a 2D differentiation system. The qRT‐PCR results showed that *WDR36* knockdown led to significant downregulation of glycolysis‐related genes, including *HK1*, *ENO1, PKM*, and *LDHA* (Figure 6G). However, western blot analysis revealed that only PKM and LDHA protein levels were significantly downregulated following *WDR36* knockdown (Figure 6H‐I). Enzyme activity assays further confirmed that *WDR36* knockdown significantly decreased PKM and LDH enzyme activity (Figure 6J). Additionally, co‐immunoprecipitation (co‐IP) results indicated that WDR36 could interact with glycolytic enzyme LDHA (Figure 6K).

In summary, these studies suggest that *WDR36* likely regulates embryonic development by affecting glycolysis‐associated enzyme activity within the glycolysis pathway. Moreover, an interaction between WDR36 and LDHA protein likely influences the specification of the TE lineage in human blastoids.

## 3 Discussion

Previous studies have demonstrated that homozygous *WDR36*‐deficient mouse embryos undergo degeneration prior to reaching blastocyst stage.^[^ ^6^ ^]^ Despite these findings, the mechanisms underlying the early blastocyst development failure associated with *WDR36* deletion have not been elucidated. In this study, we investigate the effects of *WDR36* loss‐of‐function on mouse natural embryos and blastoid models derived from human PSCs. Our observations indicate that *WDR36* interference blocks human blastoid formation, particularly impairing the commitment to the TE lineage. Targeted metabolomics analysis revealed that *WDR36* interference also reduced the production of glucose and lactate. Subsequently, we demonstrated that *WDR36* can regulate glycolytic enzyme LDHA, thereby positively influencing the differentiation of the TE lineage during the late stage of human blastocyst development.

Naturally, the development of the human embryo occurs within the maternal body, rendering direct study challenge. Recent progresses in stem‐cell‐based embryo models have facilitated new research opportunities using human PSCs.^[^ ^9^, ^10^ ^]^ When we explored the role of *WDR36* in early embryonic development using a human PSCs derived blastoid model, we found that *WDR36* interference affected the activation of YAP1 in early aggregates (Figure 1G‐H). During early embryonic development, non‐polarized inner cells exclude YAP1 from the nuclei through phosphorylation of serine127 within the Hippo signaling pathway. This exclusion results in the downregulation of GATA3 expression and an increase in OCT4 protein levels, thereby maintaining the pluripotency of inner cells and positioning them as precursors to the ICM. By contrast, in polarized outer cells, the presence of the polar region and YAP1 nuclear activity leads to the upregulation of *GATA3* expression.^[^ ^24^ ^]^ In our study, we observed that *WDR36* interference disrupts polarization and TE lineage differentiation in day 3 aggregates, as well as the final impairment of lineage differentiation in day 6 spheroids (Figures 1 and 2). Previous research has identified WDR36 as a functional homolog of yeast Utp21.^[^ ^7^ ^]^ Specifically, the loss of *WDR36* in zebrafish causes defects in 18S rRNA processing.^[^ ^5^ ^]^ Notably, studies on yeast translational regulation have highlighted the presence of numerous fragments in the leader sequence of *YAP1* mRNA that are complementary to yeast 18S rRNA, facilitating the translation process.^[^ ^25^ ^]^ Therefore, the observed reduction in nuclear localization of YAP1 due to *WDR36* interference may be related to these translation mechanisms. Additionally, despite outer cells fail to exhibit polarization, GATA3 positive cells emerge at the periphery of the day 3 aggregates (Figure 1H). This suggests that while cell polarization may enhance the expression of TE‐associated transcription factors, it is not a prerequisite for the initiation of their expression, which is consistent with the findings of Marius et al.^[^ ^26^ ^]^ and Zhu et al.^[^ ^15^ ^]^ in their studies on human embryos. This may partly explain when these polarization‐deficient aggregates were shifted to a normal culture system, although the differentiation of TE lineage decreased on day 3, they still achieved proper lineage separation on day 6 (Figure 3; Figure S4, Supporting Information).

During early embryonic development, the pluripotent ICM and TE possess different regulatory dynamics, which specify the expression patterns of lineage‐related genes in both types of cells. In this paper, we also found that *WDR36* interference mainly impacts the differentiation of the TE lineage (Figures 3 and 4). Previous studies in mice have shown that other members of the WD repeat protein family, such as *WDR74* and *WDR82*, are also involved in the specification of blastocyst lineages.^[^ ^3^, ^27^ ^]^ Specifically, knockdown of *WDR74* results in embryos that develop normally until the morula stage but fail to properly specify the ICM and TE,^[^ ^3d^ ^]^ similar to our observations in both mouse natural embryos (Figure S1, Supporting Information) and human blastoids/spheroids derived from hPSCs (Figure 3; Figure S6, Supporting Information). It is well‐known that translation regulation is critical for early mammalian embryonic development, and ribosomes play a crucial role in this process. Consequently, ensuring the normal function of ribosomes is indispensable for proper early embryonic development.^[^ ^28^ ^]^ In addition, it has been reported that *WDR36* participates in ribosome synthesis and processing.^[^ ^29^ ^]^ Our study revealed that *WDR36* interference significantly down‐regulates pathways related to ribosome synthesis, as evidenced by transcriptomics analysis, which may be associated with the observed reduction in ribosome synthesis accompanying the disruption of lineage specification.^[^ ^30^ ^]^

An increasing number of studies have highlighted the pivotal role of cellular metabolism in signal transduction and cell fate determination.^[^ ^31^ ^]^ Glycolysis, the metabolic process that converts glucose into energy and metabolites necessary for macromolecular biogenesis, has been found to regulate TE from the totipotent blastomeres during mammalian embryogenesis.^[^ ^20c^ ^]^ Furthermore, it has been shown that members of the WD repeating protein family are known to involve in the regulation of glycolysis. For example, *WDR82* facilitates the establishment of mouse iPS cells by modulating mitochondrial oxidative phosphorylation and glycolysis.^[^ ^32^ ^]^ Similarly, WD repeat‐containing protein 7 (FBW7) enhances aerobic glycolysis in pancreatic cancer cells by regulating the expression of cMYC.^[^ ^33^ ^]^ Our findings demonstrated that *WDR36* absence disturbs glucose metabolism during the late formation stage of blastoids. GO enrichment analysis and Compass prediction of cellular metabolic status revealed that *WDR36* interference significantly downregulates multiple glycolytic reactions, leading to a marked reduction in glucose and lactate concentrations (Figure 5). Hexokinase (HK) catalyzes the conversion of glucose into glucose‐6‐phosphate, which is the first rate‐limiting step of glycolysis. Glucose‐6‐phosphate can then serve as a substrate for the pentose phosphate pathway (PPP) to produce ribulose 5‐phosphate, which is crucial for generating nucleotide and glycosylation precursors involved in TE cell fate determination.^[^ ^20c^ ^]^ In contrast, we found that *WDR36* interference causes a down‐regulation of *HK1* and *HK2* mRNA levels but does not significant change their protein levels, which ultimately led to no significant changes in glucose‐6‐phosphate concentrations (Figure 5). Nonetheless, interference of *WDR36* dose affect cell fate determination, especially downregulating TE lineage specification. This may be linked to abnormal lactate levels, as we observed a significant decrease in lactate concentrations, and the co‐IP assay indicated that *WDR36* might interact with lactate dehydrogenase A (LDHA) protein. Lactate, produced by the LDHA‐catalyzed conversion of pyruvate during the later stage of glycolysis reaction, is critical for various homeostatic and pathological processes, as evidenced by the Warburg effect.^[^ ^34^ ^]^ Research using the human HTR‐8/SVneo trophoblast cell line shows that LDHA deficiency inhibits trophoblast proliferation via the PI3K/AKT/FOXO1/CyclinD1 signaling pathway.^[^ ^35^ ^]^ However, further investigations are needed to explore the unique role of *WDR36* in regulating lactate anabolism during early embryonic cell lineage specification. Furthermore, we have also found that WDR36 affects PKM protein in addition to LDHA, but how it co‐participates in the regulation of embryonic development requires further investigation.

In summary, the utilization of human blastoids derived from hPSCs presents a valuable model for studying human early embryonic development and the pathological mechanisms implicated in blastocyst arrest. Our study elucidates the crucial role of WDR36 in regulating glycolysis within human blastocyst development. These findings are pivotal in enhancing our understanding of how the regulation of pathogenic genes and glucose metabolism affects early blastocyst development, thereby offering valuable insights into infertility diseases and identifying potential therapeutic targets.

## 4 Experimental Section

### Mouse In Vitro Fertilization and Embryo Culture

All animal experiments obtained approval (Permit Number: IACUC‐2105004) from the Ethics Committee of the Animal Core Facility of Nanjing Medical University (Nanjing, China) and were performed according to institutional guidelines. Female mice were superovulated by intraperitoneal administrations of 7 IU Pregnant Mare Serum Gonadotropin (PMSG), followed 7 IU human Chorionic Gonadotropin (hCG) 48 h later. Cumulus‐oocyte complexes (COC) were isolated from the oviduct ampullae 14–15 h post‐hCG injection and cultured in droplets of fertilization medium. Sperms were collected from the tail of epididymis of adult male mice and capacitated in fertilization medium for 1 h. The capacitated spermatozoon was then introduced to the fertilization medium droplets containing COC for 6 h at 37 °C, 5% CO_2_. Finally, the zygotes were washed and transferred into droplets of KSOM medium (Aibei Biotechnology, Nanjing, M1450) for further culture.

### Generation of WDR36‐Interfered Zygotes

All siRNA sequences used in this study were listed in Table S1 (Supporting Information). Individual siRNA was diluted with water to give a working concentration of 20 µM, and ≈5 pl of the solution was injected with the Narishige microinjector. Non‐target siRNA with the same volume was injected as control. After injection, embryos were cultured in KSOM for further experiments.

### Generation of 8CLCs

Primed human UE017C1 iPS cells (gifted from Prof. Jinguang Pan) and hEPS1 cells (gifted from Prof. Hongdui Deng) were cultured in the Essential 8 (Thermo Scientific, A1517001) medium under 20% O_2_ and 5% CO_2_ at 37 °C. They were both cultured on matrigel (Corning, 354 230) coated plates and used as the starting cells to obtain 8CLCs. To generate 8CLCs, we modified the method described in the previously reported article.^[^ ^10^ ^]^ Briefly, primed human iPS cells or EPS cells were dissociated into single cells using TrypLE (Thermo Scientific, 12 604 021). These cells were plated at a density of 1000 to 1500 cells cm^−2^ on feeders in Essential 8 medium, supplemented with 10 µM Y‐27632 (selleck, S1049). 24 h later, the medium was switched to 8CLC medium, which composed of N2B27 basal medium, supplement with 100 µg mL^−1^ Vitamin C, 50 ng mL^−1^ BSA, 20 ng mL^−1^ IL6 (APExBIO, PH1001), 20 ng mL^−1^ IL‐6R (novoprotein, C691), 5 ng mL^−1^ BMP4 (StemImmune, HST‐B4‐0100) and a chemical cocktail of 0.05 µM DZNep (Cayman Chemical, 13 828), 1 µM PD0325901 (APExBIO, A3013), 1 µM CGP77675 (Cayman Chemical, 77 675), 2.5 µM XAV939 (selleck, S1180), 1µM Go6983 (APExBIO, A8343), and 5 µM Y‐27632. The 8CLC medium was refreshed daily, and the cells were passaged as single cells (at a ratio of 1:5 to 1:8) every 3 to 4 days. The formula of N2B27 basal medium was consistent with that previously reported.^[^ ^36^ ^]^

### Establishment of Doxycycline‐Inducible WDR36‐Modified Cell Lines

For generating doxycycline‐inducible *WDR36*‐interfered (i*WDR36*) iPS cell lines, we refer to previously published work in our laboratory.^[^ ^13^ ^]^ The mutually priming oligonucleotides used in this study are listed in Table S1 (Supporting Information). By using the Lipofectamine Stem Transfection Reagent (Thermo Scientific, STEM00001) according to the manufactory's instruction, the *WDR36* shRNA and pCyL43 (PiggyBac transposase) vectors were co‐transfected into iPS cells to make the inducible i*WDR36* cell lines. Puromycin (0.25 µg mL^−1^) was added for positive clone selection for 7 days, and the expanded clones were designated as the knockdown iPS cells. The expression level of *WDR36* was verified by quantitative RT‐PCR (qRT‐PCR) and immunofluorescent staining. For doxycycline‐inducible i*WDR36* hEPS1 cell lines, we used the cell lines in the previous published article for further experiment.^[^ ^13^ ^]^

### Induction of Human Blastoids

The protocol of Harunobu et al.^[^ ^9e^ ^]^ was followed to generate 8CLCs‐originated blastoids. 8CLCs were dissociated with TrypLE and removed their co‐cultured feeder cells. The cells were then resuspended in N2B27 media containing 10 µM Y‐27632 (aggregation medium) and ≈3×10^4^ cells were seeded into a well of the 24‐well of AggreWell 400 (STEMCELL Technologies, 34 450) on day 0. After cells formed aggregates within the microwell (about 24 h), the aggregation medium was replaced with PALLY medium‐N2B27 supplemented with PD0325901 (1 µM), A 83‐01 (1 µM, selleck, S7692), 1‐Oleoyl lysophosphatidic acid sodium salt (LPA) (500nM, Tocris, 3854), hLIF (10 ng mL^−1^, peprotech, 300‐05‐25UG), and Y‐27632 (10 µM). On day 3, the PALLY medium was replaced with N2B27 basal medium containing 500 nM LPA and 10 µM Y‐27632.

### Supplementation of Glucose or Lactate During the Late Formation Stage of Blastoid

8CLCs were resuspended in RPMI 1640 basic medium (Invitrogen, C11875500BT, 11.1 mM of glucose) containing 10 µM Y27632 and seeded into AggreWell plate, designating as day 0. On day 1, the medium was replaced with RPMI 1640 basic medium supplemented with PD0325901 (1 µM), A 83‐01 (1 µM), LPA (500 nM), hLIF (10 ng mL^−1^), and Y‐27632 (10 µM). From day 3 onwards, only LPA and Y‐27632 were maintained (Control Group). Building on this, additional treatments were introduced at the same timepoint: Dox for the Late iWDR36 Group, Dox and glucose for the Late iWDR36+Glucose Group, and Dox and lactate for the Late iWDR36+Lactate Group. The concentration of glucose (HUSHI, 63 005 518) used in this rescue experiment was 3 mg mL^−1^, and that of lactate (Sigma Aldrich, L4263) was 10 mM.

### RNA Isolation and qRT‐PCR Analysis

Total RNA was extracted from cells using the Trizol Reagent (Invitrogen 15596‐026) following the manufacturer's instructions, and then RNA was reverse transcribed to cDNA using a PrimeScript™ RT reagent kit with gDNA Eraser (Takara, Dalian, China). The qRT‐PCR was performed in a Step One Plus Real‐Time PCR System (Applied BioSystems, CA, USA), using the FastStart Universal SYBR Green Master kit (Vazyme, Nanjing, China). Primers were designed with Primer 5 software and their sequences were listed in Table S2 (Supporting Information). The melting curve of each mRNA was used to evaluate the amplification quality. The expression data was assessed by the 2^−△△CT^ method and the expression level of *β‐ACTIN* was used as an endogenous normalization control.

### Immunofluorescent Staining

For cell immunostaining, cells were fixed with 4% paraformaldehyde for 20 min at room temperature (RT), then permeabilized and blocked with PBS containing (vol/vol) 0.25% Triton X‐100 (Beyotime, P0096) together with (vol/vol) 2.5% donkey serum (Jackson Immuno Research, 017‐000‐121) for 60 min at RT. The primary antibody, as detailed in Table S3 (Supporting Information), was diluted in the blocking solution and the cell samples were incubated overnight at 4 °C. Subsequently, the samples were washed 5 times with PBS for 3 min each. The secondary antibodies, also specified in Table S3 (Supporting Information), were diluted in 2.5% donkey serum and incubated at RT for 1 h. Finally, the samples underwent 3 additional PBS washes, and the nuclei were stained with 4′,6‐diamidino‐2‐phenylindole (DAPI) (YIFEIXUE BIO TECH, YD0020‐10). Mouse morula, E3.5 embryos, and blastoids were fixed in 4% PFA for 30 min at RT, and then permeabilized in 0.2% TritonX‐100 overnight at 4 °C, and blocked with blocking buffer (2.5% donkey serum, 0.1% Tween‐20 and 0.1% BSA in PBS) overnight at 4 °C. Primary antibodies were diluted in blocking buffer and incubated overnight at 4 °C. The embryos were washed in a washing solution (PBS with 0.1% BSA) for three times. Secondary antibodies were diluted in a washing solution. The embryos or blastoids were incubated with secondary antibodies for 1 h in the dark at room temperature. The nuclei were stained with DAPI for 10 min. The immunostaining images were collected by utilizing a confocal microscopy (ZEISS LSM700, Germany).

### Co‐Immunoprecipitation (Co‐IP) Assay

The interaction between WDR36 and LDHA was assessed using a Co‐IP assay kit (Beyotime, P2179) according to the manufacturer's protocol. First, cells were lysed in lysis buffer supplemented with protease inhibitors. Subsequently, 250 µL of cell lysates containing 100 µg Protein A + G magnetic beads were supplemented with antibodies against WDR36, LDHA, or IgG as the negative control, and a certain proportion of supernatant without any antibody (Input) was used as the positive control, followed by incubation for 2 h, with tumbling, at room temperature. The magnetic beads were separated via magnetic force followed by washing with ice‐cold lysis buffer. Subsequently, the magnetic beads were immersed in SDS‐PAGE sample loading buffer and boiled for 5 min. Following magnetic separation for 10 s, the supernatant was collected for western blotting, using the aforementioned method.

### Western Blot Analysis

Total protein was extracted from cells using radio immunoprecipitation assay (RIPA) lysis buffer (Beyotime, P0013B). The solution was centrifuged at 4 °C, 20 000 × g for 10 min, and the supernatant was then collected for the following step. The protein samples were mixed with sodium dodecyl sulfate‐polyacrylamide gel (SDS‐PAGE) protein loading buffer (5 ×). The protein samples were denatured at a high temperature of 100 °C for 5 min. Equal amounts of proteins were separated using an 8%‐12% SDS‐PAGE. After electrophoresis, the protein was transferred to a polyvinylidene difluoride membrane (Biosharp, BS‐PVDF‐22). 5% BSA was used to seal the membrane for 1 h. The membrane was then incubated with the primary antibodies overnight and the secondary antibodies for 2 h. For imaging, membranes were incubated with the ECL Ultra Western HRP Substrate (Vazyme, E412‐01) and revealed by an imaging system (ChemiDoc XRS, Bio‐Rad). The antibody information can be found in Table S3 (Supporting Information).

### PKM and LDH Enzymatic Activity Assays

Samples were collected and lysed in extracting solution. After centrifugation at 8000g at 4 °C for 10 min, the supernatant was collected and placed on ice for measurement. A pyruvate kinase assay kit (Solarbio, BC0540) and lacate dehydrogenase (LDH) activity assay kit (Solarbio, BC0685) was used to determine PKM and LDH enzymatic activity, respectively. The working solution was real‐time‐prepared according to manufactures’ protocol. The change in absorbance was measured at 340 nm (450 nm for LDH enzymatic activity) wavelength.

### RNA Sequencing (RNA‐Seq)

We constructed the RNA‐seq libraries following the SMART‐seq2 protocol.^[^ ^37^ ^]^ Briefly, in the first step, each sample was stored in 2 µL lysis buffer (0.2% Triton X‐100 and 40 U mL^−1^ Recombinant RNase Inhibitor). After incubating the samples in 10 µL Oligo(dT) buffer at 72 °C for 3 min, the tubes were immediately put back on ice for 2 min. Then, 4 µL of 5x RT buffer and 4 µL Reverse Transcriptase buffer (ABclonal, RK20310) were added to each sample for first‐strand cDNA synthesis, followed by cDNA amplification using Amplification Module PCR Mix (1 µL PCR primer and 29 µL PCR Master Mix) for 16 cycles. Thereafter, the library was purified with 1x DNA Clean beads (Vazyme, N411‐01). Tn5 Enzyme mix and PCR mix (ABclonal, RK20237) were used for tagmentation to construct sequencing libraries from the amplified cDNA. Finally, the DNA library amplification was performed by adding 25 µL PCR Mix and 5 µL amplification primers (10 µM) to the products, followed by library purification with 0.6x and 0.15x DNA Clean beads (Vazyme, N411‐01). The quality of the libraries for sequencing was verified with PerkinElmer LabChip GX Touch. Sequencing was performed on the Illumina NovaSeq 6000 platform in the PE150 mode.

### RNA‐Seq Data Preprocessing

The RNA‐seq data were first subjected to adaptor trimming and low‐quality read filtering with flexbar (version 2.5)^[^ ^38^ ^]^ with the following parameters: ‐u 6 ‐m 36 ‐ae RIGHT ‐at 2 ‐ao 2 ‐x 3 ‐y 1. The trimmed paired‐end reads were then aligned to the human reference genome (hg19) by STAR (version 2.7.10b)^[^ ^39^ ^]^ under the parameters: ‐outFilterMismatchNoverLmax 0.1 –alignIntronMin 20 –alignIntronMax 1 000 000 –alignSJoverhangMin 6 –alignSJDBoverhangMin 1 –outFilterType BySJout –outFilterIntronMotifs RemoveNoncanonicalUnannotated, followed by gene expression quantification by HTSeq (v0.13.5).^[^ ^40^ ^]^ Fragments per kilobase of transcript per million fragments mapped (FPKM) values of genes were calculated as $\mathrm{FPK}\;M_{g}=\frac{C_{g}}{l_{g}\sum_{i}C_{i}}\times 10^{9}$, where C_g_ denotes the fragment count of gene g, and l_g_ is the total non‐redundant exonic length of gene g.

### Principal Component Analysis (PCA)

The transcriptomic data of blastoids generated in this study was compared to human natural blastocysts and blastoids from previously published studies.^[^ ^9^, ^17^ ^]^ Taking account of the differences in RNA‐seq protocols, gene expression values were converted into transcripts per million (TPM). For 10x Genomics single‐cell data, TPM values were directly defined as $TP\;M_{g}=\frac{C_{g}}{\sum_{i}C_{i}}\;\times 10^{6}$, where *C_g_* denotes the read count of gene *g*. For bulk RNA‐seq and Smart‐Seq2 data, TPM values were calculated as $TP\;M_{g}=\frac{FPKM_{g}}{\sum_{i}FPKM_{i}}\times 10^{6}$. To infer the gene expression profiles at the lineage level, the machine‐learning method CIBERSORTx^[^ ^41^ ^]^ was used. Briefly, the EPI, HYPO, and TE expressions of blastoids were taken by Kagawa et al.^[^ ^9e^ ^]^ as the reference expression signatures, then the expression levels measured in bulk were mathematically separated into lineage‐specific profiles CIBERSORTx. Subsequently, the inferred expression values were log2 transformed and PCA was performed using the prcomp function in R with following parameters: center = T, scale = T.

### Differential Expression Analysis

To identify differentially expressed genes (DEG), lowly expressed genes (i.e., sum of counts in all compared samples below 10) were first excluded, and then applied the R package DESeq2 (version 1.30.1)^[^ ^42^ ^]^ for analysis. Significant DEGs were defined as genes of adjusted p‐value < 0.05 and log2FoldChange > 1 (upregulated) or log2FoldChange < ‐1 (downregulated). Functional enrichment analysis of DEGs was performed using enrichGO and enrichKEGG functions of the R package clusterProfiler (version 4.8.3).^[^ ^43^ ^]^ Gene‐set enrichment analysis (GSEA) was also performed using the R package clusterProfiler on the gene lists ranked by fold changes.

### Targeted Metabolomics Analysis

Targeted metabolomics analysis followed the procedure reported previously.^[^ ^44^ ^]^ In detail, fifty blastoids or spheroids were washed 3 times with cold PBS and transferred to a 1.5 mL Eppendorf tube under a stereoscopic microscope. Cell pellet was then flash frozen in liquid nitrogen to quench enzyme activity and stop metabolite degradation. Subsequently, 400 µL of methanol: H_2_O (80:20, v/v, precooled at −80 °C) containing an internal standard (i.e., acetaminophen) was added to the tube, and the cells were fully lysed by using an ultrasonic cell disruptor (Bioruptor Pico, Belgium) for 20 min at 4 °C. The tube was vortexed vigorously for 5 min, and centrifuged at 16 000 × *g* for 10 min at 4 °C to remove cell debris. Afterwards, the metabolite‐containing supernatant was transferred to a new tube on ice. The supernatant was then dried down using a vacuum concentrator (Labconco, USA) and reconstituted with 80 µL of methanol: H_2_O (1:1, v/v). The solution was sonicated for 10 min, followed by centrifugation at 20 000 x g for 15 min at 4 °C to remove insoluble debris. Finally, the supernatant was transferred to a sample vial for subsequent targeted metabolomics analysis. Quantification of cellular metabolites was performed by using an ExionLC AD30 system (AB SCIEX, USA) and a QTRAP 6500+ mass spectrometer (AB SCIEX, USA). Multiple reaction monitoring (MRM) transitions were optimized for each analyte (glucose *m/z* 179.0 → *m/z* 70.9, *m/z* 179.0 → *m/z* 88.9, *m/z* 179.0 → *m/z* 119.1, lactate *m/z* 89.0 → *m/z* 43.0, *m/z* 89.0 → *m/z* 45.1, glucose 6‐phosphate *m/z* 259.1 → *m/z* 78.8, *m/z* 259.1 → *m/z* 96.8, acetaminophen *m/z* 150.0 → *m/z* 107.0, *m/z* 150.0 → *m/z* 131.9). In brief, 5 µL of sample was loaded onto a 100×2.1 mm Kinetex EVO C18 column (Phenomenex, USA). The mobile phase consisted of phase A (10 mM ammonium acetate and 10 mM ammonium hydroxide in water) and phase B (acetonitrile), with a flow rate of 0.3 mL min^−1^. The metabolites were eluted with a gradient program as follows: 0 min (40% B) → 1 min (40% B) → 2 min (60% B) → 3 min (60% B) → 4 min (40% B) → 5 min (40% B). The column chamber temperature was set to 40 °C. The ion source settings in negative mode analysis were as follows: curtain gas (CUR) = 35, ion spray voltage (IS) = ‐4500, TEM = 450, ion source gas 1 (GS1) = 50 and ion source gas 2 (GS2) = 50. AB SCIEX Analyst software v1.6.3 (AB SCIEX, USA) was used for data collection and management. The peak areas of the target metabolite transitions and internal standard transitions were separately summed. The metabolite areas were normalized to the internal standard for the following calculation. The surrogate matrix used for the preparation of calibration standards in this study was BSA solution at an approximate concentration of 0.5 mg mL^−1^. The suitability of this matrix has been carefully evaluated.^[^ ^45^ ^]^ Then, the limit of detection (LOD), limit of quantification (LOQ) and linear range of each metabolite were determined. The calibration curves were obtained by plotting the relative peak area ratios of the target metabolites and the internal standard against the concentration of the target metabolites. The linear ranges were determined as follows: glucose, 50–4000 ng mL^−1^; lactate, 100–4000 ng mL^−1^; and glucose 6‐phosphate, 50–4000 ng mL^−1^. Quality controls (QCs), which included the lower limit of quantification (LLOQ), low QC, mid QC and high QC, were employed for accuracy and precision validation. As a result, both the precision (coefficient of variation, CV) and accuracy (%bias) for the target metabolites were acceptable (≤20% for LLOQ, ≤15% for other QCs).

### Statistical Analysis

All data were presented as mean ± S.D. Statistical analysis was performed using the two‐tailed Student's *t*‐test, Fisher's exact test or one‐way analysis of variance (ANOVA) and was defined as statistically significant when *P* < 0.05.

## Conflict of Interest

The authors declare no conflict of interest.

## Author Contributions

S.A., S.H., F.X., and H.Y. contributed equally to this work. Conceptualization: Y.Y., X.W., Y.C., and S.A., Methodology: S.A., S.H., and F.X., Investigation: S.A., S.H., W.Z., H.Y., H.C., L.D., and X.S., Data curation: S.H. and H.Y., Visualization: J.X., Supervision and Funding acquisition: Y.Y., Writing‐original draft: S.A., Y.Y., and X.W., Writing‐review & editing: Y.Y., X.W., and Y.C.

## Supporting information



Supporting Information

## Data Availability

The data that support the findings of this study are available in the supplementary material of this article.
