# Supplementary material for: WDR36 Regulates Trophectoderm Differentiation During Human Preimplantation Embryonic Development Through Glycolytic Metabolism
Source: Adv Sci (Weinh). 2024 Dec 10;12(5):2412222. doi: 10.1002/advs.202412222 (PMC11791977; doi:10.1002/advs.202412222)
Supplement: Supplementary file 1 — Supporting Information [file ADVS-12-2412222-s001.docx]

Supporting Information

*WDR36* regulates trophectoderm differentiation during human preimplantation embryonic development through glycolytic metabolism

*Shiyu An^1†^, Shuyue Hou^1†^, Feifei Xu^2†^, Huanyu Yan^1†^, Wenyi Zhang^1^, Jinfeng Xiang^3, 4^, Haoran Chen^2^, Hanwen Zhang^1^, Lingling Dong^1^, Xiaobin Sun^1^, Ran Huo^1^, Yun Chen ^1, 2*^, Xi Wang^1,5*^, Yang Yang^1,6*^*

^1^ State Key Laboratory of Reproductive Medicine and Offspring Health, Nanjing Medical University, Nanjing, China, 211166.

^2^ School of Pharmacy, Nanjing Medical University, Nanjing, China.

^3^ Fourth Clinical Medicine College, Nanjing Medical University, Nanjing, China.

^4^ Department of Obstetrics, Women’s Hospital of Nanjing Medical University, Nanjing Maternity and Child Health Care Institute, Nanjing, China.

^5^ Department of Prenatal Diagnosis of the First Affiliated Hospital of Nanjing Medical University, Nanjing, China.

^6^ Innovation Center of Suzhou Nanjing Medical University, Suzhou, 215000, Jiangsu, China

^†^ These authors contributed equally to this work.

***Corresponding author:**

**Yang Yang**: yangyang11@njmu.edu.cn

**Xi Wang**: xiwang@njmu.edu.cn

**Yun Chen**: ychen@njmu.edu.cn

This PDF file includes:

Figures. S1 to S10

Tables S1 to S3

Data S1


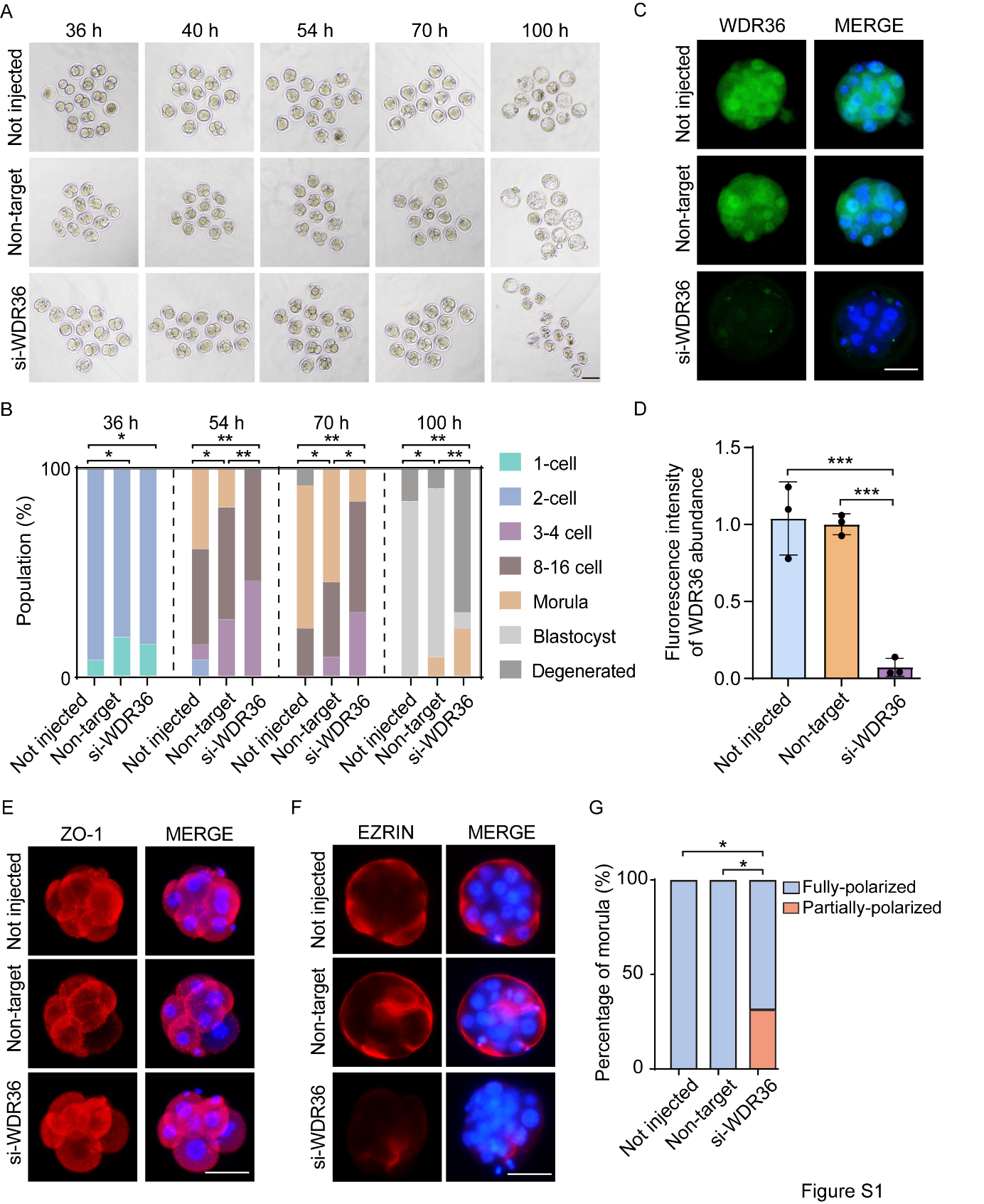


Fig. S1. Depletion of *WDR36* mRNA leads to preimplantation embryonic arrest in mouse.

A. Phase-contrast images of mouse embryo without injection and injected with siRNA (non-target siRNA and si-WDR36) at 36 h, 40 h, 54 h, 70 h and 100 h. Bar =100 μm.

B. Stacked bar plots display the distribution of the not-injected, non-target siRNA injected and si-WDR36 injected embryos at different developmental stages. The number of embryos from not injected group, non-target siRNA group and si-WDR36 group were 13,11 and 14, respectively. Data were represented three independent experiments and Fisher’s exact test were used for statistical analysis. *, *P* < 0.05, **, *P* < 0.01, and no labelling indicated no significance.

C. Representative immunofluorescence images showing the expression of WDR36 in mouse morula in different groups. Bar =50 μm.

D. The relative fluorescence intensity of WDR36 abundance was analyzed from (C). Data were represented three independent experiments. The results were presented as mean ± S.D., and analyzed by one-way ANOVA. ***, *P* < 0.001, and no labelling indicated no significance.

E-F. Representative immunofluorescence images of morula to reveal the localization of ZO-1 (E) and EZRIN (F). Bar =50 μm.

G. Percentages of fully polarized, partially-polarized and non-polarized cell aggregates in each group. n = 10 morula embryos for each group. Data were represented three independent experiments and Fisher’s exact test were used for statistical analysis. * *P* < 0.05.


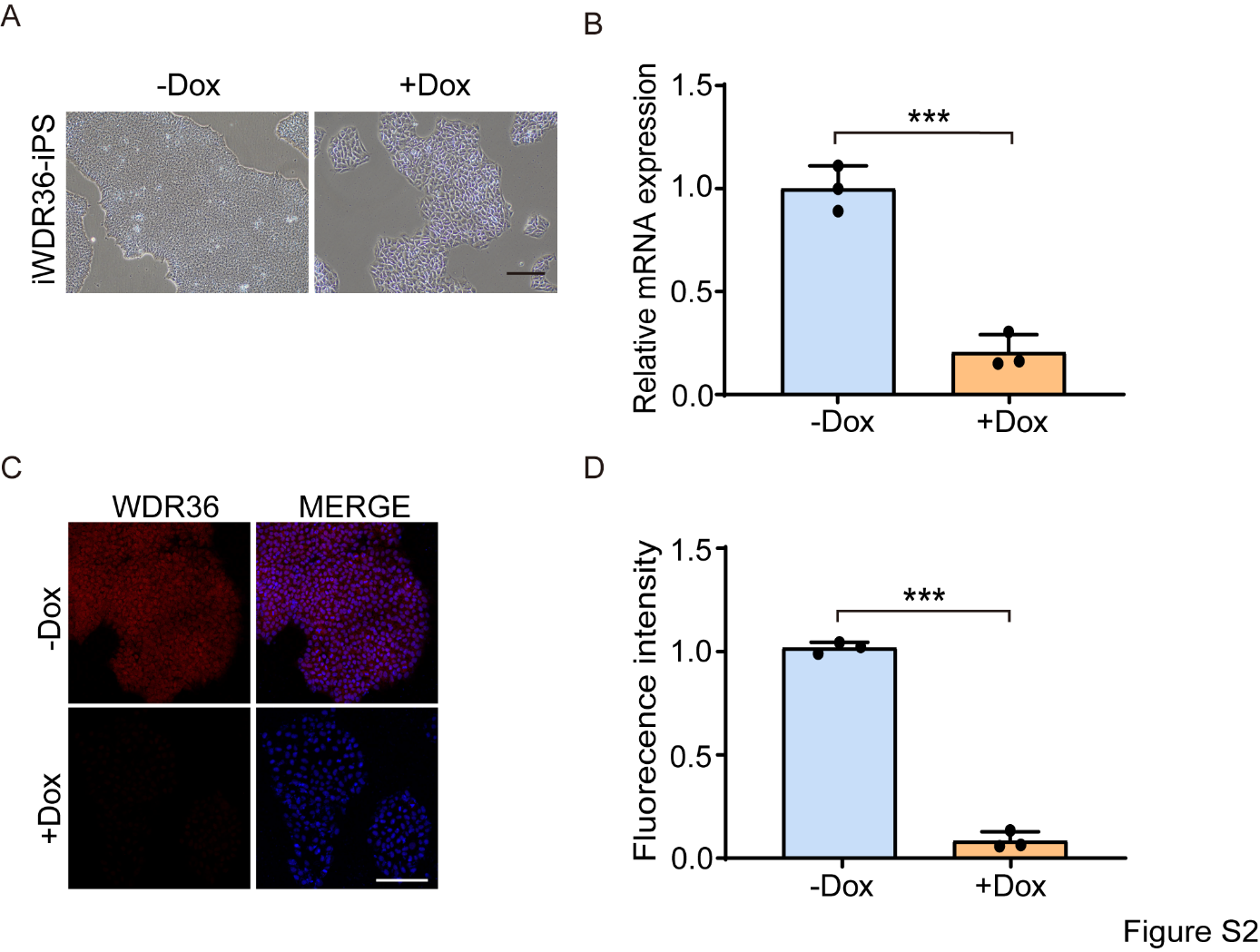


Fig. S2. Characteristics of iWDR36 iPS.

A. Representative phase-contrast images of iWDR36 iPS colonies. Bar =100 μm.

B. The mRNA expression level of *WDR36* in iWDR36 iPS cultured in different conditons. Data were represented as mean ± S.D. in three independent experiments and two-tailed Student’s t-test were used for statistical analysis. *** *P* < 0.001.

C-D. Immunostaining and statistical analysis for expression levels of WDR36 in iWDR36 iPS. Bar =100 μm. Data were represented as mean ± S.D. in three independent experiments and two-tailed Student’s t-test were used for statistical analysis. *** *P* < 0.001.


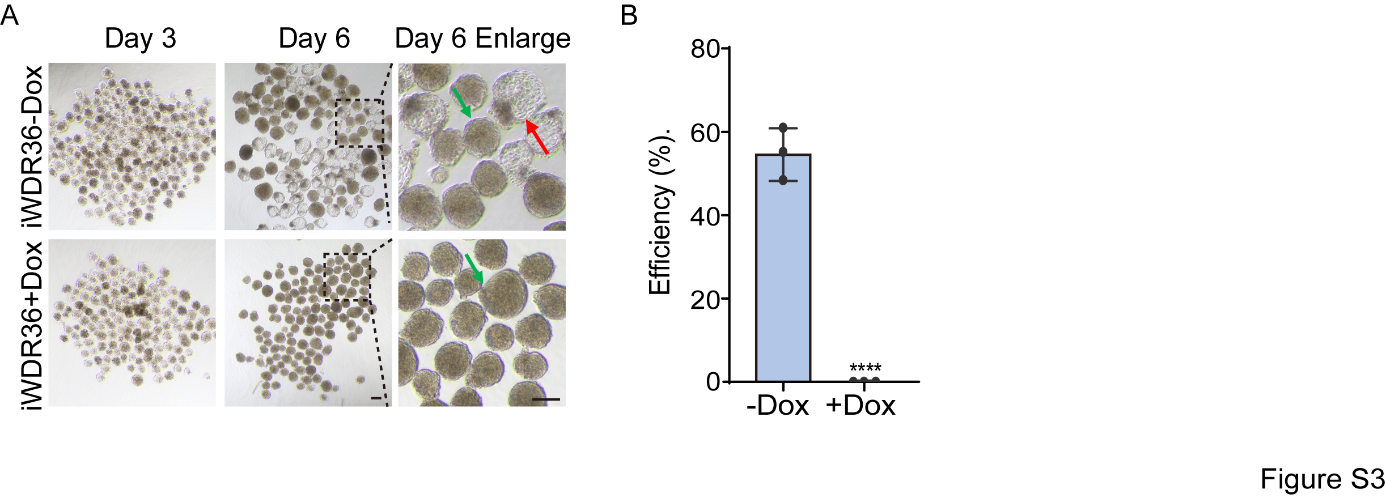


Fig. S3. *WDR36* inhibition hinders cavity-like structures formation potential of human EPS1-derived 8CLCs.

A. Phase-contrast images of cell aggregates at different timepoints when they were cultured in the indicated conditions. Bars =100 μm. Red arrowheads indicate blastoid, green arrowheads indicate spheroid.

B. Quantification of blastoids formation efficiency for hEPS1-derived 8CLCs cultured in the indicated conditions. Data were represented as mean ± S.D. in three independent experiments and two-tailed Student’s t-test were used for statistical analysis. **** *P* < 0.0001.


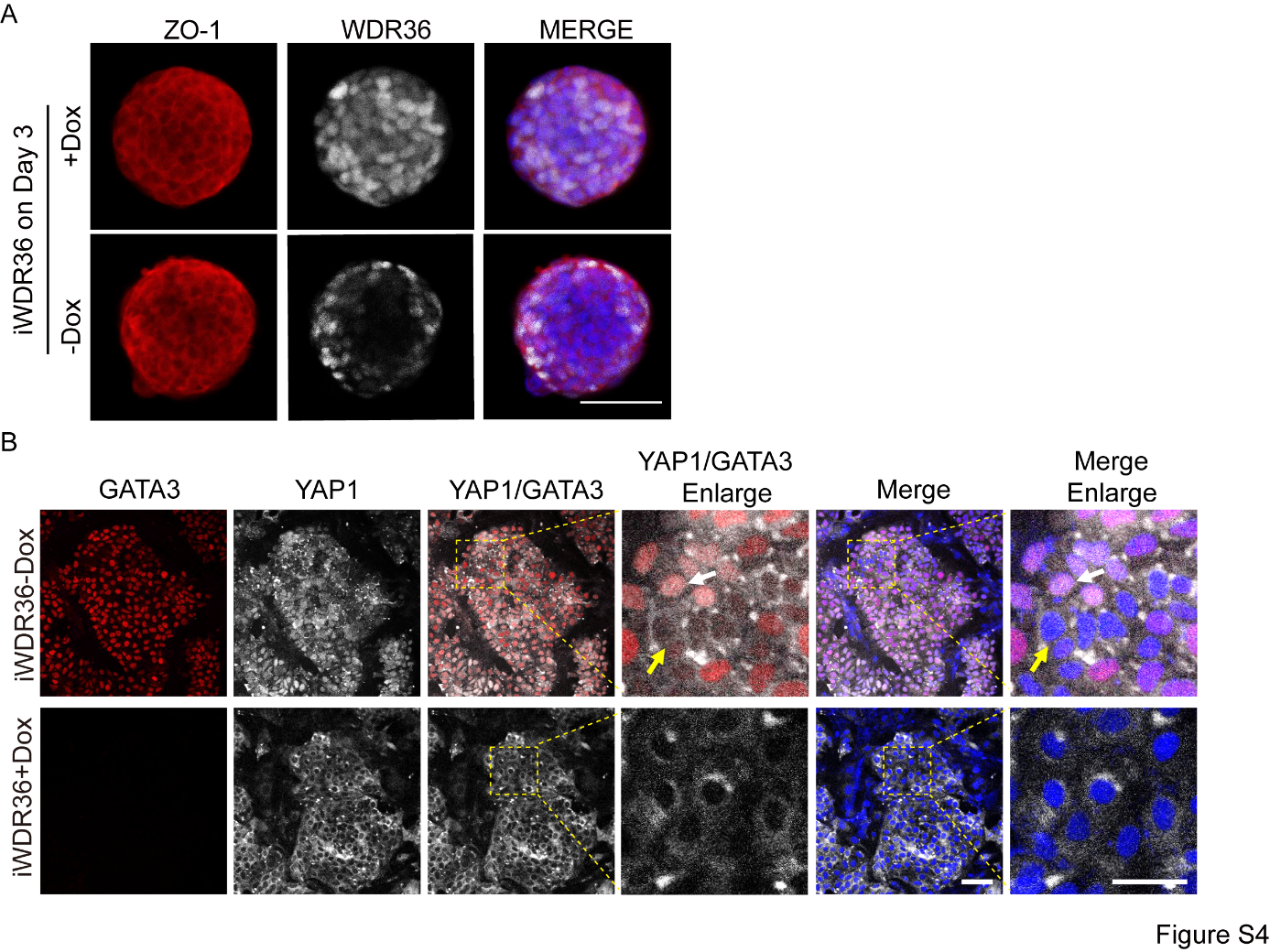


Fig. S4. Further characteristics of iPS-8CLCs-aggregates and the differentiation potential assessment of iPS-8CLCs in a 2D differentiation assay.

A. Representative immunofluorescence co-staining images of ZO-1 and WDR36 in day 3’s aggregates, with and without WDR36 interference. Bar =50 μm.

B. Representative immunofluorescence co-staining images of GATA3 and YAP1 on the day 5 of 8CLCs differentiation toward the trophoblast, with or without WDR36 interference. Scale bars, 50 μm. The yellow arrows highlight cytoplasm-localized YAP1, and the white arrows show nucleus-localized YAP1.


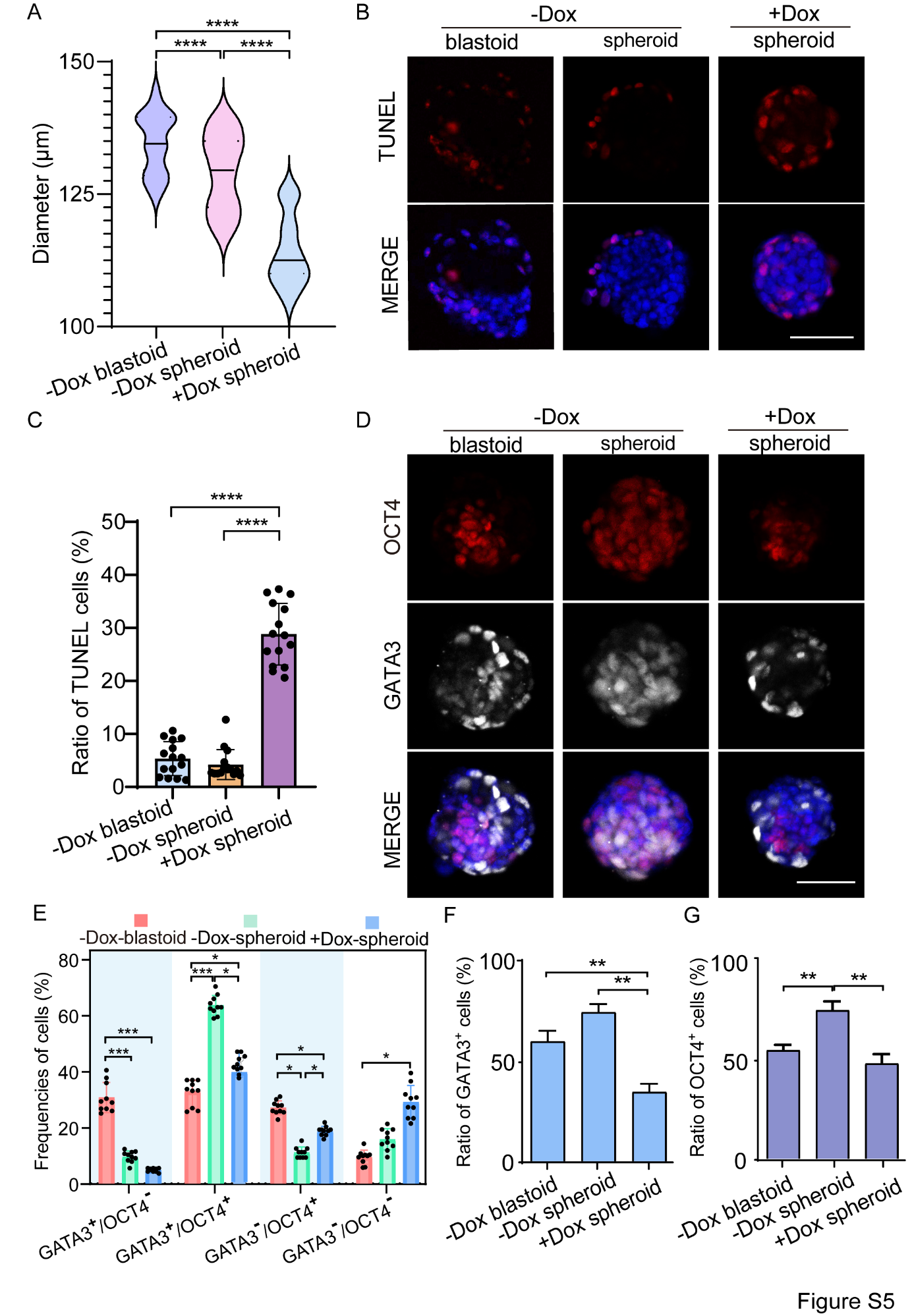


Fig. S5. Interfering *WDR36* throughout the blastoid formation process disrupts self-organization potential of human EPS1-derived 8CLCs.

A. Quantification of the average diameters of blastoids and spheroids. N = 35 blastoids or 35 spheroids. Data were represented as mean ± S.D. in three independent experiments and one-way ANOVA were used for statistical analysis. **** *P* < 0.0001.

B. Immunofluorescent images of the blastoids and spheroids to visualize apoptosis (TUNEL). Bar =100 μm.

C. The apoptotic ratio (apoptotic cell number/total cell number) of each blastoid or spheroid. N = 15 blastoids or spheroids. Data were represented as mean ± S.D. in three independent experiments and one-way ANOVA were used for statistical analysis. **** *P* < 0.0001. No labeling indicates no statistical significance.

D. Immunostaining for lineage-specific markers in the blastoids and spheroids (GATA3 for TE, OCT4 for ICM). Bar =100 μm.

E. Percentage of GATA3^+^/OCT4^-^, GATA3^+^/OCT4^+^, GATA3^-^/OCT4^+^ and GATA3^-^/OCT4^+^ cells in each blastoid/spheroid in the -Dox and +Dox groups. N = 15 blastoids or spheroids. Data were represented as mean ± S.D. in three independent experiments and one-way ANOVA were used for statistical analysis. * *P* < 0.05. *** *P* < 0.001. No labeling indicates no statistical significance.

F-G. Quantification of the percentage of GATA3^+^ and OCT4^+^ cells from (G). N = 15 blastoids or spheroids. Data were represented as mean ± S.D. in three independent experiments and one-way ANOVA were used for statistical analysis. ** *P* < 0.01. No labeling indicates no statistical significance.


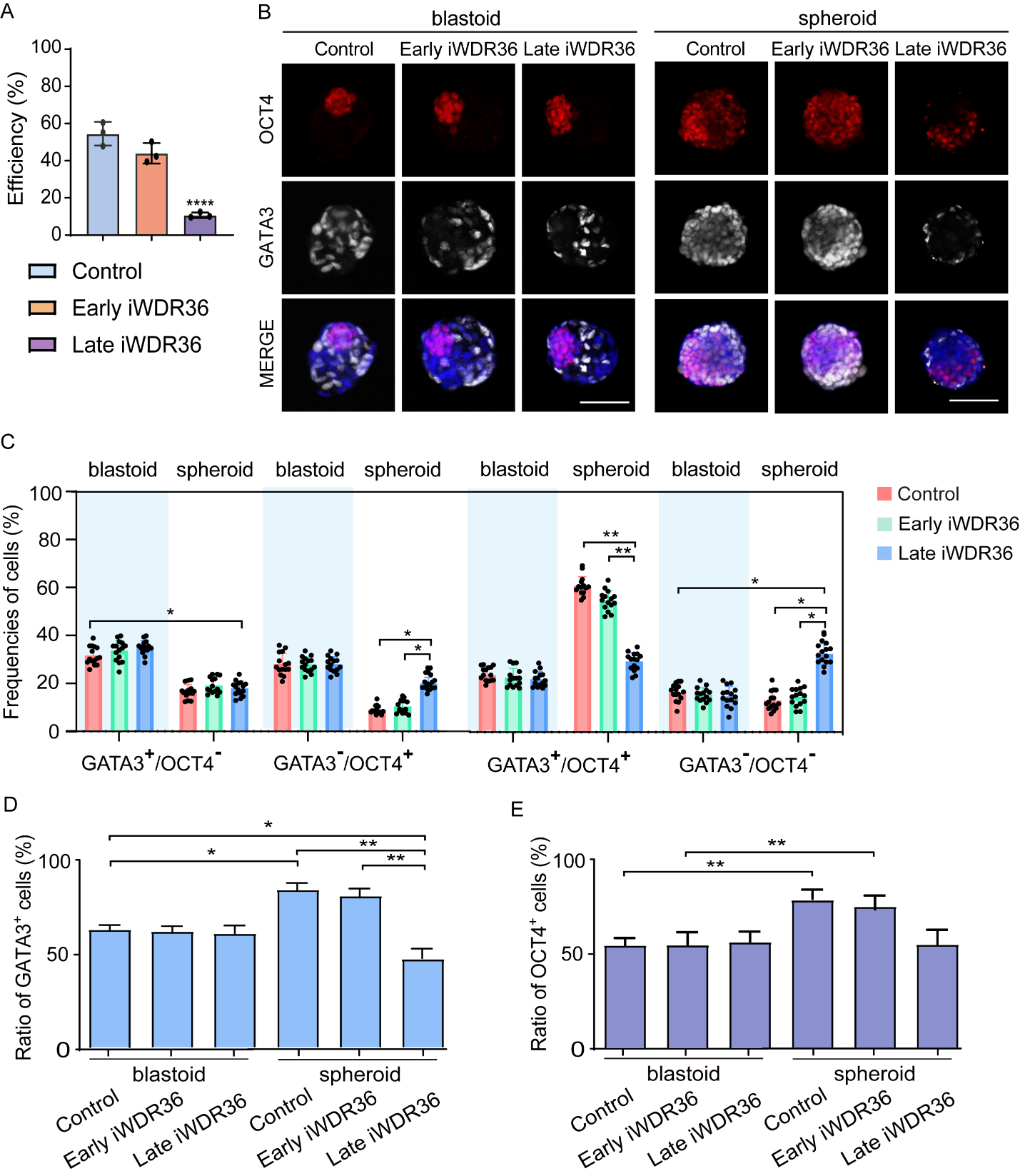


Fig. S6. *WDR36* interference mainly hinders the late stage of EPS-blastoids formation.

A. Quantification of blastoids formation efficiency when *WDR36* interference were conducted at different stages.

B. Immunofluorescent images of blastoids/spheroids for visualizing lineage differentiation (GATA3 for TE, OCT4 for ICM). Bar =100 μm.

C. Frequencies of different cell types in each blastoid or spheroid. N = 15 blastoids or spheroids. Data were represented as mean ± S.D. in three independent experiments and one-way ANOVA were used for statistical analysis. No labeling indicates no statistical significance.

D-E Percentages of GATA3^+^ and OCT4^+^ cells from (C). N = 15 blastoids/spheroids. Data were represented as mean ± S.D. in three independent experiments and one-way ANOVA were used for statistical analysis. * P < 0.05, ** P < 0.01. No labeling indicates no statistical significance.


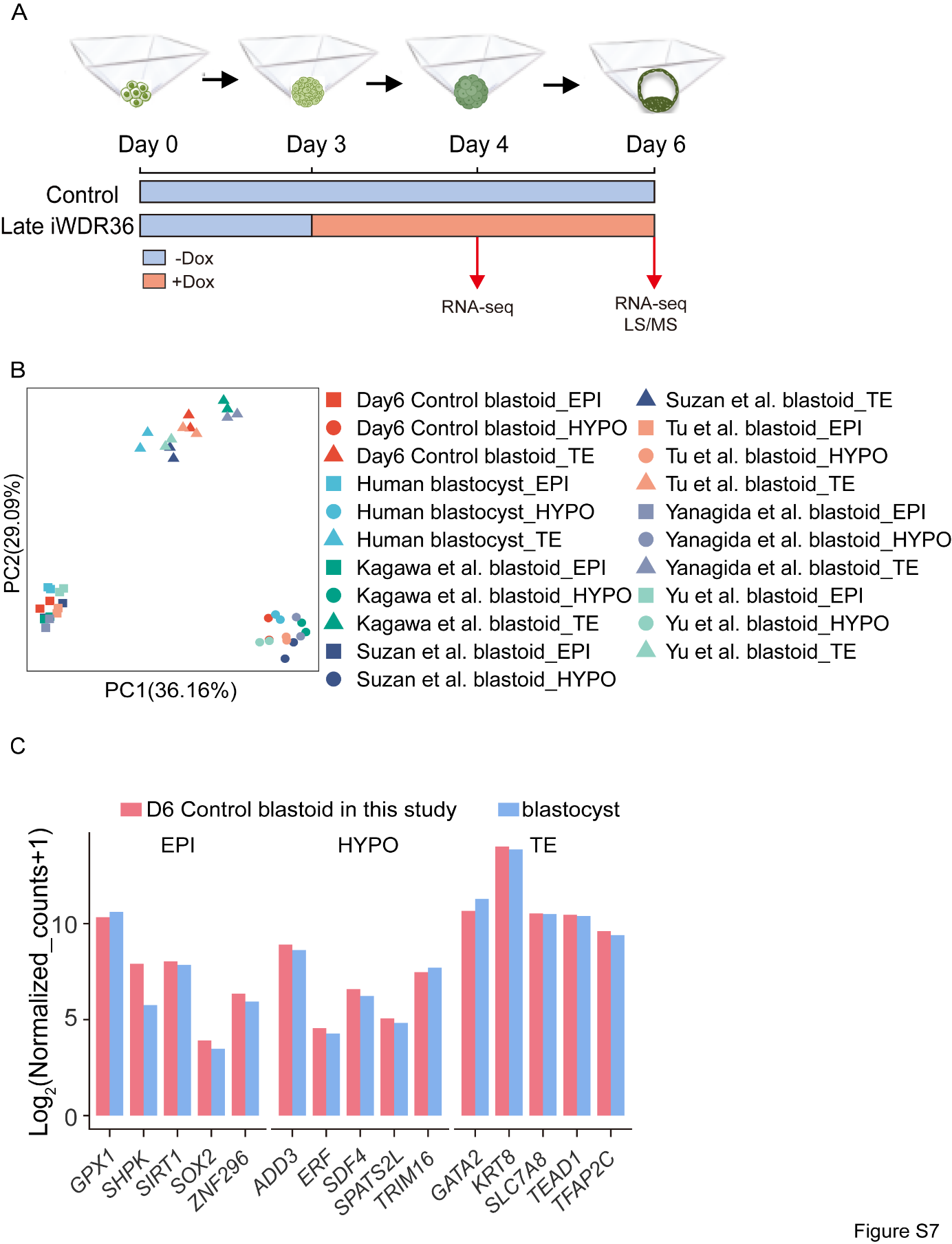


Fig. S7. *In vitro* cultured iPS-8CLCs-blastoids exhibits similar transcriptional profiles to multiple originated blastoids and natural human blastocyst.

A. A diagram showing sample collection and analyses for bulk RNA-seq and LS/MS.

B. PCA analysis comparing linage-specific expression profiles of the Day 6 Control blastoid (red label) in this study, blastoids in other studies and human natural blastocysts.

C. The expression of representative genes for EPI, TE and HYPO lineages in D6 Control blastoids and blastocysts. D6 means day 6.


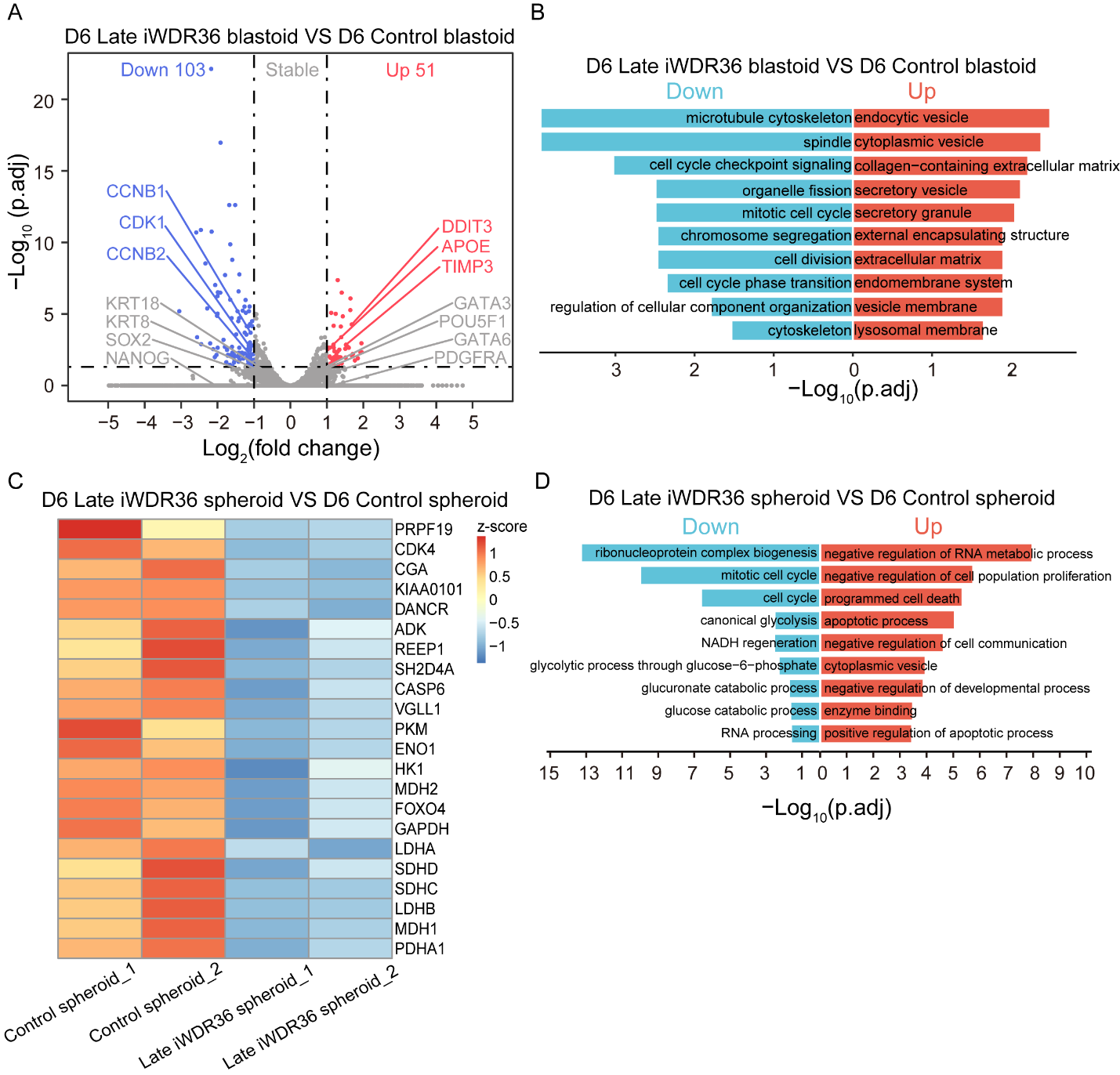


Fig. S8. Transcriptome analysis for impact of *WDR36* on the formation of blastoid and spheroid structures.

A. Volcano plots for the globally transcriptional differences between D6 Late iWDR36 blastoid and D6 Control blastoid. Red dots indicated up-regulated (FC > 2) and blue dots indicated down-regulated (FC < -2) genes with adjusted *P* < 0.05. Grey dots indicated genes with no significant difference with either adjusted *P* ≥ 0.05 or absolute value of FC ≤ 2.

B. Enrichment of GO terms of up-regulated (red) and down-regulated (blue) genes in the comparison of D6 Late iWDR36 blastoid and D6 Control blastoid.

C. Heatmap of representative marker genes for glycolysis and TE differentiation in the comparison of D6 Late iWDR36 spheroid and D6 Control spheroid.

D. Enrichment of GO terms of up-regulated (red) and down-regulated (blue) genes in D6 Late iWDR36 spheroid compared to D6 Control spheroid.

D6 means day 6.


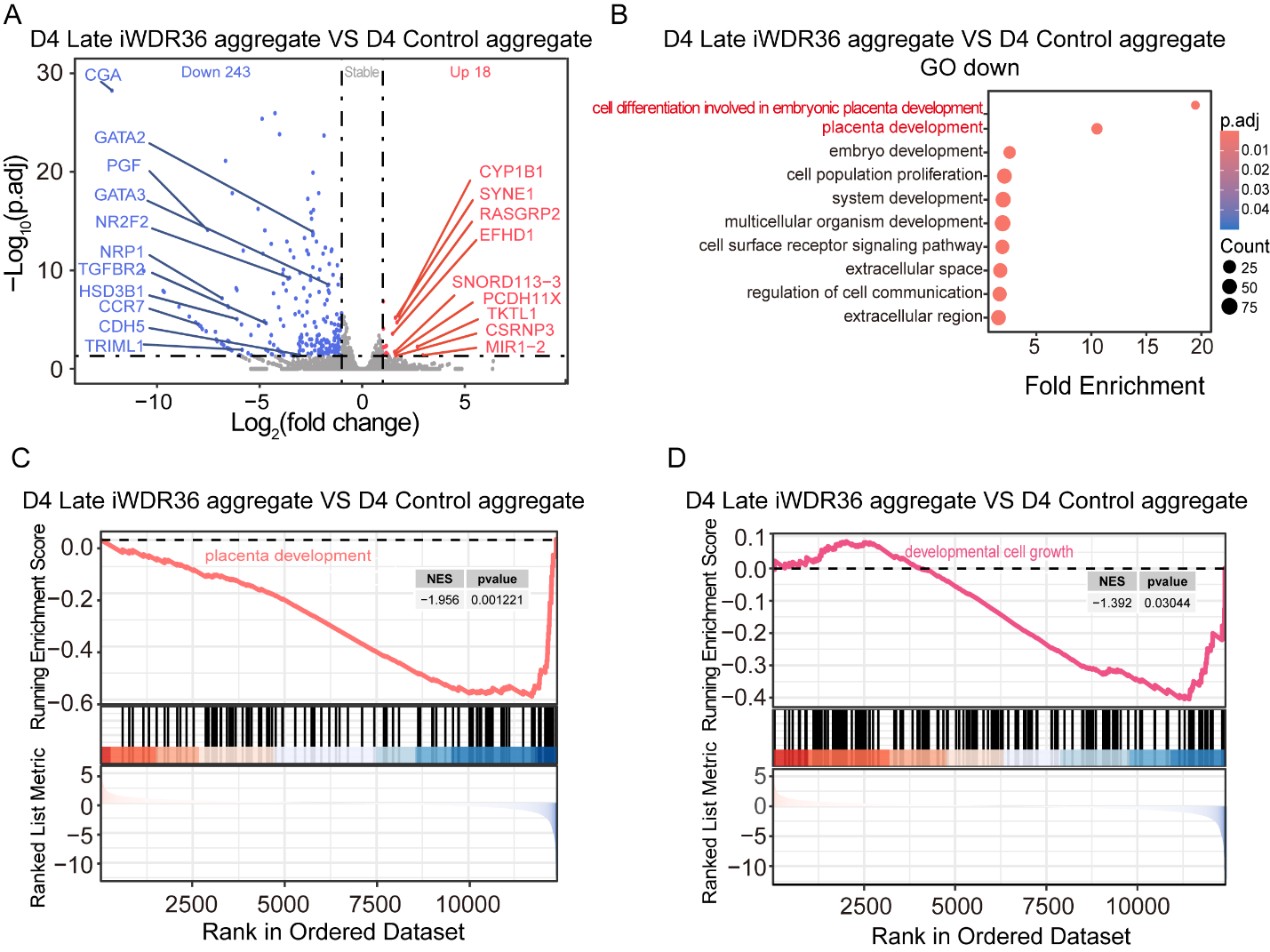


Fig. S9. Transcriptomic analysis of the effects of *WDR36* on the D4 aggregates.

A. Volcano plots for the globally transcriptional differences between D4 Late iWDR36 aggregate and D4 Control aggregate. Red dots indicated up-regulated (FC > 2) and blue dots indicated down-regulated (FC < -2) genes with adjusted P < 0.05. Grey dots indicated genes with no significant difference with either adjusted P ≥ 0.05 or absolute value of FC ≤ 2.

B. Dotplot showed enriched GO terms of down-regulated genes in the comparison of D4 Late iWDR36 aggregate and D4 Control aggregate.

C-D. GSEA analysis of placenta development and developmental cell growth related genes in D4 Late iWDR36 aggregate compared to D4 Control aggregate. Normalized enrichment score (NES) and nominal p values were shown.

D4 means day 4.


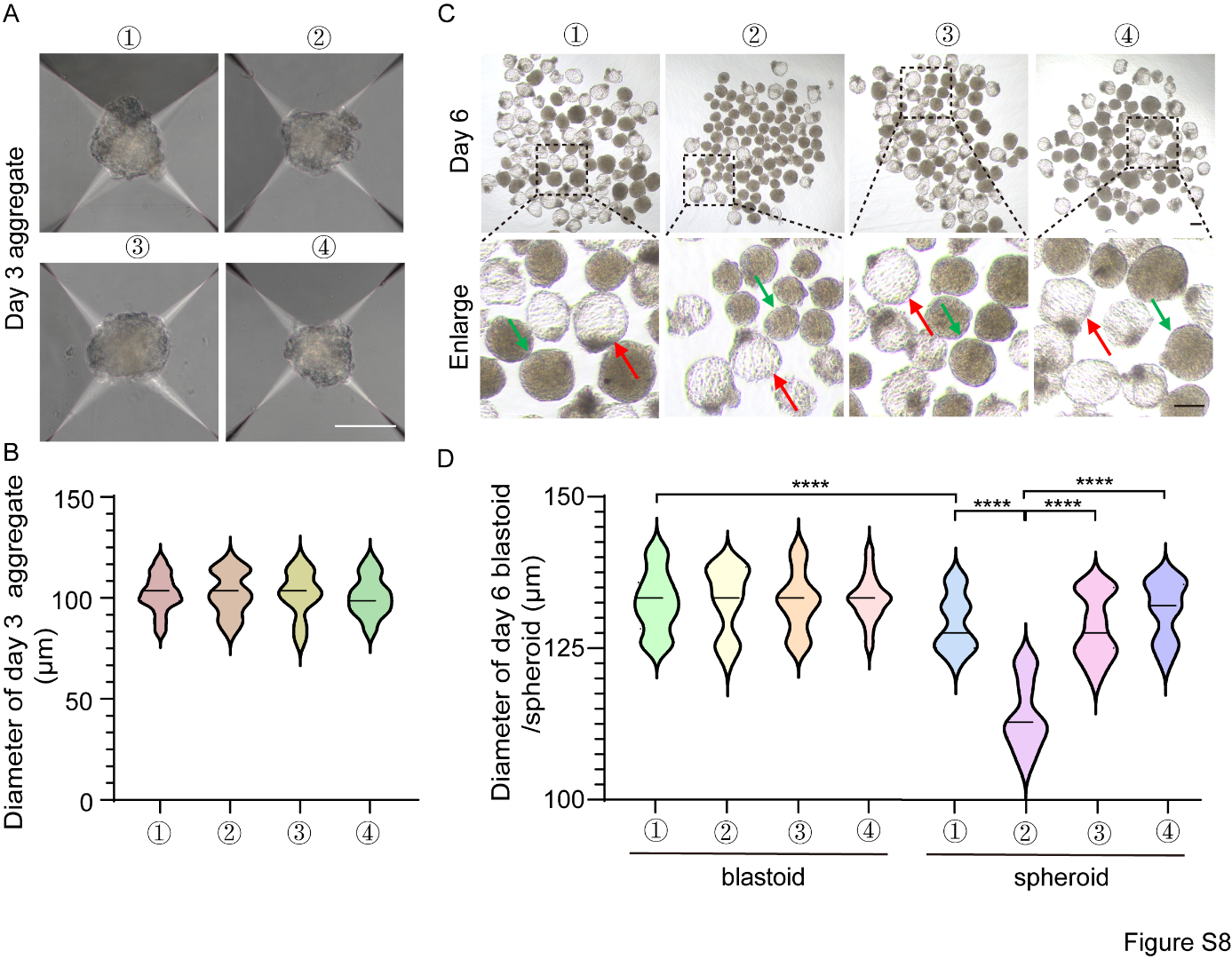


Fig. S10. Morphological features for iPS-8CLCs originated aggregates (day 3) and blastoids/spheroids (day 6).

A. Phase-contrast images of cell aggregates at day 3 when they were cultured in the indicated conditions. Bar =100 μm.

B. Quantification of the diameters of iPS-8CLCs originated aggregates on day 3. Data were represented as mean ± S.D. in three independent experiments and one-way ANOVA were used for statistical analysis. No labeling indicates no statistical significance.

C. Phase-contrast images of blastoids/spheroids at day 6 when they were cultured in the indicated conditions. Bars =100 μm. Red arrows indicate blastoid, green arrows indicate spheroid.

D. Quantification of the diameters of blastoids and spheroids on day 6. Data were represented as mean ± S.D. in three independent experiments and one-way ANOVA were used for statistical analysis. **** *P* < 0.0001. No labeling indicates no statistical significance.

After iPS-8CLCs were seeded, they were randomly cataloged into four groups, including: ①, -Dox, ②, +Dox, ③, +Dox+Glucose, ④, +Dox+Lactate.

Table S1. Primer sequences of siRNA and shRNA in this study.

| Name | Sequence (5′- 3′) |
| --- | --- |
| siRNA-scramble | F: UUCUCCGAACGUGUCACGUTT  R: ACGUGACACGUUCGGAGAATT |
| siRNA-WDR36 | F: GCCGUGGAUGUUGUUGCUAUUTT  R: AAUAGCAACAACAUCCACGGCTT |
| shRNA-scramble | GTTCTCCGAACGTGTCACGT |
| shRNA-WDR36 | CCGGGCCGTGGATGTTGTTGCTATTCTCGAGAATAGCAACAACATCCACGGCTTTTTT |

Table S2. Details of primer sequences of genes used for qPCR.

| **Primers** | **Sequences** (5'-3') |
| --- | --- |
| *WDR36*-F  *WDR36*-R | TGGCAAGGATCAAGCTCACAA  AAGTCATCCAAGGCGAGTCC |
| *HK1-F*  *HK1-R* | GCTCTCCGATGAAACTCTCATAG |
|  | GGACCTTACGAATGTTGGCAA |
| *HK2-F* | TGCCACCAGACTAAACTAGACG |
| *HK2-R* | CCCGTGCCCACAATGAGAC |
| *ENO1-F*  *ENO1-R* | AAAGCTGGTGCCGTTGAGAA |
|  | GGTTGTGGTAAACCTCTGCTC |
| *MYC-F*  *MYC-R* | TCCCTCCACTCGGAAGGAC |
|  | CTGGTGCATTTTCGGTTGTTG |
| *PKM-F* | ATAACGCCTACATGGAAAAGTGT |
| *PKM-R* | TAAGCCCATCATCCACGTAGA |
| *LDHA-F*  *LDHA-R* | ATGGCAACTCTAAAGGATCAGC |
|  | CCAACCCCAACAACTGTAATCT |
| *LDHB-F* | ACCAGGCCCTACTTGTCCTT |
| *LDHB-R* | TTCATCAGCCAGAGACTTTCCC |
| *SLC2A1-F* | GGCCAAGAGTGTGCTAAAGAA |
| *SLC2A1-R* | ACAGCGTTGATGCCAGACAG |
| *β-ACTIN-F*  *β-ACTIN-R* | CATGTACGTTGCTATCCAGGC |
|  | CTCCTTAATGTCACGCACGAT |
| *GAPDH-F*  *GAPDH-R* | TCGACAGTCAGCCGCATCTTCTTT  ACCAAATCCGTTGACTCCGACCTT |

Table S3. Details of antibodies used in this study.

| **Products** | **Cat.** | **Sources** | **Diluted Ratios** |
| --- | --- | --- | --- |
| Rabbit anti-WDR36 | ab73548 | Abcam | 1:200 |
| Rabbit Anti-GATA3 | ab181557 | Abcam | 1:1000 |
| Mouse Anti-Oct-3/4 (C-10) | sc-5279 | Santa Cruz | 1:500 |
| Rabbit Anti-ZO-1 | 5406S | CST | 1:500 |
| Rabbit Anti-p-Ezrin/ERM | 3726S | CST | 1:500 |
| Mouse Anti-PAR6 | sc-166405 | Santa Cruz | 1:500 |
| Rabbit Anti-YAP1 | 13584-1-AP | proteintech | 1:500 |
| Rabbit Anti-HK1 | 2024S | CST | 1:1000 |
| Mouse Anti-ZO-1 | 66452-1-Ig | proteintech | 1:500 |
| Rabbit Anti-ENO1 | A1033 | Abclone | 1:500 |
| Rabbit Anti-PKM | A13905 | Abclone | 1:500 |
| Rabbit Anti-LDHA | 19987-1-AP | proteintech | 1:1000 |
| Rabbit Anti-β-ACTIN | BS6007M | Bioworld | 1:20000 |
| HRP-conjugated Affinipure Goat Anti- Rabbit | SA00001-2 | proteintech | 1:20000 |
| HRP-conjugated Affinipure Goat Anti-Mouse | SA00001-1 | proteintech | 1:20000 |
| Donkey anti-Goat, Alexa Fluor 647 | A21447 | Thermo Fisher Scientific | 1:500 |
| Donkey anti-Mouse, Alexa Fluor 488 | A21202 | Thermo Fisher Scientific | 1:500 |
| Donkey anti-Rabbit, Alexa Fluor 555 | A31572 | Thermo Fisher Scientific | 1:500 |

Data S1. Compass analysis the differences in glycolysis metabolism between the WDR36 interference and control groups.

**Late iWDR36 spheroid VS Control blastoid**

| Reaction ID | Reaction formula | cohens_d | Corresponding description | sample_  activate |
| --- | --- | --- | --- | --- |
| \| ENO_pos \| \| --- \| \|  \| | 1.00 * D-Glycerate 2-phosphate [c] --> 1.00 * Water [c] + 1.00 * Phosphoenolpyruvate [c] | -9.07592 | Phosphoenol pyruvate | Control blastoids |
| ENO_neg | 1.00 * D-Glycerate 2-phosphate [c] --> 1.00 * Water [c] + 1.00 * Phosphoenolpyruvate [c] | -25.9166 | D-Glycerate 2-phosphate | Control blastoids |
| r0355_pos | 1.00 * dATP [c] + 1.00 * D-glucose [c] --> 1.00 * proton [c] + 1.00 * dADP [c] + 1.00 * D-Glucose 6-phosphate [c] | -14.8805 | dADP+D-Glucose 6-phosphate | Control blastoids |
| r0354_pos | 1.00 * ITP(3-) [c] + 1.00 * D-glucose [c] --> 1.00 * proton [c] + 1.00 * IDP(3-) [c] + 1.00 * D-Glucose 6-phosphate [c] | -14.8805 | IDP+D-Glucose 6-phosphate | Control blastoids |
| PGMT_neg | 1.00 * D-Glucose 1-phosphate [c] --> 1.00 * D-Glucose 6-phosphate [c] | -5.57898 | D-Glucose 1-phosphate | Control blastoids |
| DPGM_pos | 1.00 * 3-Phospho-D-glyceroyl phosphate [c] --> 1.00 * proton [c] + 1.00 * 2,3-bisphosphonato-D-glycerate(5-) [c] | -3.43124 | 2,3-bisphosphonato-D-glycerate | Control blastoids |
| r0173_neg | 1.00 * Nicotinamide adenine dinucleotide [x] + 1.00 * (S)-lactate [x] --> 1.00 * proton [x] + 1.00 * Nicotinamide adenine dinucleotide - reduced [x] + 1.00 * pyruvate [x] | -3.16414 | NADH+lactate | Control blastoids |
| PGI_neg | 1.00 * D-Glucose 6-phosphate [c] --> 1.00 * D-Fructose 6-phosphate [c] | -5.14729 | D-Glucose 6-phosphate | Control blastoids |
| PGM_pos | 1.00 * D-Glycerate 2-phosphate [c] --> 1.00 * 3-Phospho-D-glycerate [c] | -5.1004 | 3-Phospho-D-glycerate | Control blastoids |
| PGI_pos | 1.00 * D-Glucose 6-phosphate [c] --> 1.00 * D-Fructose 6-phosphate [c] | -2.79838 | D-Fructose 6-phosphate | Control blastoids |
| TPI_pos | 1.00 * Dihydroxyacetone phosphate [c] --> 1.00 * Glyceraldehyde 3-phosphate [c] | -2.36613 | Glyceraldehyde 3-phosphate | Control blastoids |
| LDH_L_neg | 1.00 * Nicotinamide adenine dinucleotide [c] + 1.00 * (S)-lactate [c] --> 1.00 * proton [c] + 1.00 * pyruvate [c] + 1.00 * Nicotinamide adenine dinucleotide - reduced [c] | -2.07059 | lactate | Control blastoids |
| FBA_neg | 1.00 * D-Fructose 1,6-bisphosphate [c] --> 1.00 * Dihydroxyacetone phosphate [c] + 1.00 * Glyceraldehyde 3-phosphate [c] | -0.0989 | D-Fructose 1,6-bisphosphate | Control blastoids |
| G6PPer_pos | 1.00 * Water [r] + 1.00 * D-Glucose 6-phosphate [r] --> 1.00 * D-glucose [r] + 1.00 * hydrogenphosphate [r] | -1.86457 | D-glucose | Control blastoids |
| TPI_neg | 1.00 * Dihydroxyacetone phosphate [c] --> 1.00 * Glyceraldehyde 3-phosphate [c] | 0.801641 | Dihydroxyacetone phosphate | Late iWDR36 spheroids |
| ALDD2x_pos | 1.00 * Water [c] + 1.00 * Nicotinamide adenine dinucleotide [c] + 1.00 * acetaldehyde [c] --> 2.00 * proton [c] + 1.00 * Nicotinamide adenine dinucleotide - reduced [c] + 1.00 * acetate [c] | 1.117386 | NADH+acetate | Late iWDR36 spheroids |

**Late iWDR36 spheroid VS Control spheroid**

| Reaction ID | Reaction formula | cohens_d | Corresponding description | sample_  activate |
| --- | --- | --- | --- | --- |
| \| PGM_pos \| \| --- \| | 1.00 * D-Glycerate 2-phosphate [c] --> 1.00 * 3-Phospho-D-glycerate [c] | -2.6885 | 3-Phospho-D-glycerate | Control spheroids |
| GAPD_pos | 1.00 * hydrogenphosphate [c] + 1.00 * Nicotinamide adenine dinucleotide [c] + 1.00 * Glyceraldehyde 3-phosphate [c] --> 1.00 * proton [c] + 1.00 * Nicotinamide adenine dinucleotide - reduced [c] + 1.00 * 3-Phospho-D-glyceroyl phosphate [c] | -2.13042 | NADH+3-Phospho-D-glyceroyl phosphate | Control spheroids |
| G6PPer_pos | 1.00 * Water [r] + 1.00 * D-Glucose 6-phosphate [r] --> 1.00 * D-glucose [r] + 1.00 * hydrogenphosphate [r] | -0.50749 | D-glucose  +hydrogenphosphate | Control spheroids |
| ENO_neg | 1.00 * D-Glycerate 2-phosphate [c] --> 1.00 * Water [c] + 1.00 * Phosphoenolpyruvate [c] | -1.5272 | D-Glycerate 2-phosphate | Control spheroids |
| PGI_neg | 1.00 * D-Glucose 6-phosphate [c] --> 1.00 * D-Fructose 6-phosphate [c] | -0.57722 | D-Glucose 6-phosphate | Control spheroids |
| r0354_pos | 1.00 * ITP(3-) [c] + 1.00 * D-glucose [c] --> 1.00 * proton [c] + 1.00 * IDP(3-) [c] + 1.00 * D-Glucose 6-phosphate [c] | -1.49306 | IDP+D-Glucose 6-phosphate | Control spheroids |
| r0355_pos | 1.00 * dATP [c] + 1.00 * D-glucose [c] --> 1.00 * proton [c] + 1.00 * dADP [c] + 1.00 * D-Glucose 6-phosphate [c] | -1.49306 | dADP+D-Glucose 6-phosphate | Control spheroids |
| r0173_neg | 1.00 * Nicotinamide adenine dinucleotide [x] + 1.00 * (S)-lactate [x] --> 1.00 * proton [x] + 1.00 * Nicotinamide adenine dinucleotide - reduced [x] + 1.00 * pyruvate [x] | -1.29801 | NADH+lactate | Control spheroids |
| PGMT_neg | 1.00 * D-Glucose 1-phosphate [c] --> 1.00 * D-Glucose 6-phosphate [c] | -1.03299 | D-Glucose 1-phosphate | Control spheroids |
| LDH_L_neg | 1.00 * Nicotinamide adenine dinucleotide [c] + 1.00 * (S)-lactate [c] --> 1.00 * proton [c] + 1.00 * pyruvate [c] + 1.00 * Nicotinamide adenine dinucleotide - reduced [c] | -1.06972 | lactate | Control spheroids |
| ENO_pos | 1.00 * D-Glycerate 2-phosphate [c] --> 1.00 * Water [c] + 1.00 * Phosphoenolpyruvate [c] | -0.80737 | Phosphoenolpyruvate | Control spheroids |
| DPGM_pos | 1.00 * 3-Phospho-D-glyceroyl phosphate [c] --> 1.00 * proton [c] + 1.00 * 2,3-bisphosphonato-D-glycerate(5-) [c] | -0.3385 | 2,3-bisphosphonato-D-glycerate | Control spheroids |
| FBA_neg | 1.00 * D-Fructose 1,6-bisphosphate [c] --> 1.00 * Dihydroxyacetone phosphate [c] + 1.00 * Glyceraldehyde 3-phosphate [c] | -0.17139 | D-Fructose 1,6-bisphosphate | Control spheroids |
| TPI_neg | 1.00 * Dihydroxyacetone phosphate [c] --> 1.00 * Glyceraldehyde 3-phosphate [c] | 3.321635 | Dihydroxyacetone phosphate | Late iWDR36 spheroids |
| ALDD2y_pos | 1.00 * Water [c] + 1.00 * Nicotinamide adenine dinucleotide phosphate [c] + 1.00 * acetaldehyde [c] --> 2.00 * proton [c] + 1.00 * Nicotinamide adenine dinucleotide phosphate - reduced [c] + 1.00 * acetate [c] | 5.921435 | NADH+acetate | Late iWDR36 spheroids |
